# Systemic Administration of Cowpea Mosaic Virus Demonstrates Broad Protection Against Metastatic Cancers

**DOI:** 10.1002/advs.202308237

**Published:** 2024-03-02

**Authors:** Young Hun Chung, Zhongchao Zhao, Eunkyeong Jung, Anthony O. Omole, Hanyang Wang, Lucas Sutorus, Nicole F. Steinmetz

**Affiliations:** ^1^ Department of Bioengineering University of California, San Diego La Jolla CA 92093 USA; ^2^ Moores Cancer Center University of California, San Diego La Jolla CA 92093 USA; ^3^ Department of NanoEngineering University of California, San Diego La Jolla CA 92093 USA; ^4^ Center for Nano‐ImmunoEngineering University of California, San Diego La Jolla CA 92093 USA; ^5^ Department of Biology University of California, San Diego La Jolla CA 92093 USA; ^6^ Department of Radiology University of California, San Diego La Jolla CA 92093 USA; ^7^ Institute for Materials Discovery and Design University of California, San Diego La Jolla CA 92093 USA; ^8^ Center for Engineering in Cancer University of California, San Diego La Jolla CA 92093 USA; ^9^ Shu and K.C. Chien and Peter Farrell Collaboratory University of California, San Diego La Jolla CA 92093 USA

**Keywords:** adjuvant therapy, cancer, CPMV, metastasis

## Abstract

The key challenge in cancer treatment is prevention of metastatic disease which is therapeutically resistant and carries poor prognoses necessitating efficacious prophylactic approaches that prevent metastasis and recurrence. It is previously demonstrated that cowpea mosaic virus (CPMV) induces durable antitumor responses when used in situ, i.e., intratumoral injection. As a new direction, it is showed that CPMV demonstrates widespread effectiveness as an immunoprophylactic agent – potent efficacy is demonstrated in four metastatic models of colon, ovarian, melanoma, and breast cancer. Systemic administration of CPMV stimulates the innate immune system, enabling attack of cancer cells; processing of the cancer cells and associated antigens leads to systemic, durable, and adaptive antitumor immunity. Overall, CPMV demonstrated broad efficacy as an immunoprophylactic agent in the rejection of metastatic cancer.

## Graphical abstract

Systemic adjuvant therapy with immumodulatory cowpea mosaic virus provides board efficacy against metastatic disease. CPMV‐induced anti‐tumor immunity is tumor and tissue‐agnostic.

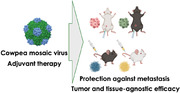

## 1 Introduction

Metastatic cancers tend to be resistant to therapeutics, more aggressive, and demonstrate increased plasticity (e.g., by instigating angiogenesis in hypoxic conditions).^[^ ^1^, ^2^, ^3^ ^]^ The difficulty in treating metastatic cancer is evidenced by the fact that 66.7 – 90% of all cancer‐related deaths are attributed to metastases.^[^ ^4^, ^5^ ^]^ Prophylactic strategies that prevent onset and outgrowth of metastatic disease and those that prevent recurrence of disease after initial treatment (termed adjuvant therapy) are urgently needed.^[^ ^6^, ^7^ ^]^ Adjuvant therapy has already proven to be extremely successful in the prevention of breast and colon cancer metastases;^[^ ^8^, ^9^ ^]^ however, the current gold standard adjuvants are combinations of chemotherapeutics, which harbor harsh side effects and reduce quality of life.^[^ ^10^ ^]^ The chemotherapeutic regimens can also be taxing and require adjuvant therapy for up to months at a time at high dose density.^[^ ^11^ ^]^ Additionally, in patients that develop metastases regardless of adjuvant therapy, the cancer acquires adjuvant chemotherapy‐induced resistance, and patient life expectancy drops compared to patients not provided any adjuvant therapy.^[^ ^12^, ^13^ ^]^

Immunotherapies are becoming standard of care in many different indications. In previous work, we demonstrated that the plant virus, cowpea mosaic virus (CPMV), is a potent immunomodulator and primes potent, systemic, and durable antitumor immunity when used as an in situ therapeutic.^[^ ^14^, ^15^, ^16^, ^17^, ^18^ ^]^ While non‐infectious in mammals, it is recognized as foreign and stimulates an innate immune response through the activation of pattern recognition receptors (PRRs) via its endogenous pathogen‐associated molecular patterns (PAMPs). Specifically, the toll‐like receptor (TLR) class of PRRs are activated with the CPMV capsid activating TLRs 2 and 4 while the RNA activates TLR7.^[^ ^19^ ^]^ The TLR activation then stimulates downstream immune signaling pathways such as NFκB that promote the activation and recruitment of immune cells, polarizes cells into antitumor phenotypes (e.g., M1 macrophage and N1 neutrophils), and releases immunostimulatory cytokines such as interferon γ (IFNγ), interleukin‐6 (IL‐6), interleukin‐12 (IL‐12), and tumor necrosis factor α (TNFα).^[^ ^17^, ^20^ ^]^ In past studies, CPMV was used primarily as an in situ vaccine with direct intratumoral (i.t.) injection into the primary tumor – we have demonstrated potent efficacy in murine models of melanoma, ovarian, colon, breast cancer, and glioma^[^ ^14^, ^15^, ^16^, ^17^, ^18^ ^]^ as well as in canine patients with melanoma and inflammatory mammary cancer.^[^ ^21^, ^22^ ^]^

Here, we demonstrate that CPMV functions as an immunoprophylactic adjuvant therapy and treatment rejects metastatic cancers in multiple murine tumor models. Longitudinal analyses demonstrate long‐lasting adjuvanticity after a single administration of CPMV and development of systemic immune memory. We utilize two different metastatic models in both BALB/C and C57BL/6J mice to demonstrate the wide scope and applicability of CPMV adjuvant therapy.

## 2 Results and Discussion

### 2.1 CPMV Production and Characterization

**Figure 1:**
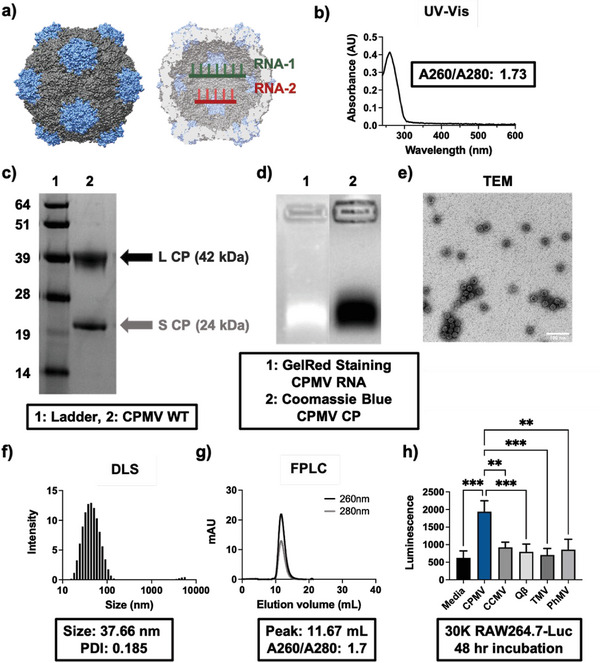
Characterization of CPMV nanoparticles. a) Structure of CPMV. CPMV consists of a bipartite genome of RNA‐1 and RNA‐2 encapsulated in identical, but separate capsids. The CPMV capsid (PDB ID: 1NY7) was reconstructed using UCSF Chimera software. b) UV–vis spectra of CPMV. The black inset of ≈1.73 indicates the A260/A280 ratio of CPMV, which is within range of the ideal value of 1.8. c) SDS‐PAGE of wild‐type (WT) CPMV showing its small (S) and large (L) CP of 24 and 42 kDa, respectively, of which it has 60 copies each. d) Agarose gel electrophoresis of CPMV. The co‐migration of the RNA and the capsid shows that the CPMV nanoparticles are intact and stable. e) TEM of CPMV. The particles are icosahedral in shape and are ≈30 nm. The scale bar represents 100 nm. f) DLS of CPMV. Size and polydispersity index (PDI) are shown. g) FPLC SEC of CPMV. The CPMV elutes at ≈11 mL from the Superose 6 Increase column, and the A260/A280 value at the peak is ≈1.7 mirroring the UV–vis data from b). h) RAW264.7‐Luc NFκB reporter assay. CPMV demonstrates unique immunostimulatory properties, while control VNPs are inert. Both Qβ and PhMV are expressed as VLPs. ** = *p* < 0.01, *** = *p* < 0.001. The data is displaying the average luminescence, and the error bars represent the standard deviation. The experiment was run in triplicate. The graph in h) was analyzed using one‐way ANOVA. CCMV = cowpea chlorotic mottle virus; Qβ is a bacteriophage, here used as a VLP; TMV = tobacco mosaic virus, PhMV = physalis mottle virus, here used as a VLP.

CPMV is a 30 nm, positive‐sense RNA plant virus with a bipartite genome (**Figure** **1**a) with two distinct RNAs (RNA‐1 and RNA‐2).^[^ ^23^ ^]^ Ultraviolet‐visible spectroscopy (UV–vis) of the CPMV shows A260/A280 values near 1.8 indicating pure particles (Figure 1b). Analysis of the proteins by sodium dodecyl sulfate‐polyacrylamide gel electrophoresis (SDS‐PAGE) shows the small (S) and large (L) coat proteins (CPs) of ≈24 and 42 kDa, respectively (Figure 1c); 60 copies each form an intact CPMV particle. Native gel electrophoresis of the CPMV shows that the genomic RNA and capsid co‐migrate indicating stable, unbroken particles (Figure 1d). Transmission electron microscopy (TEM), dynamic light scattering (DLS), and size exclusion chromatography (SEC) highlight that monodisperse and pure CPMV preparations were obtained (Figure 1e–g).

As part of the quality control and assurance, we confirmed the immunostimulatory nature of CPMV and also compared it to other viral nanoparticles (VNPs) using a RAW264.7 macrophage NFκB luminescent reporter cell line (RAW264.7‐Luc). While CPMV showed significant luminescent signal, indicating innate immune stimulation over the media‐only control, the other VNPs did not elicit stimulation (Figure 1h). This confirms the unique nature of CPMV in its immunostimulatory capabilities.^[^ ^24^ ^]^

### 2.2 CPMV Adjuvant Therapy Against CT26‐Luc Colon Cancer Disseminated in the i.p. Space

**Figure 2:**
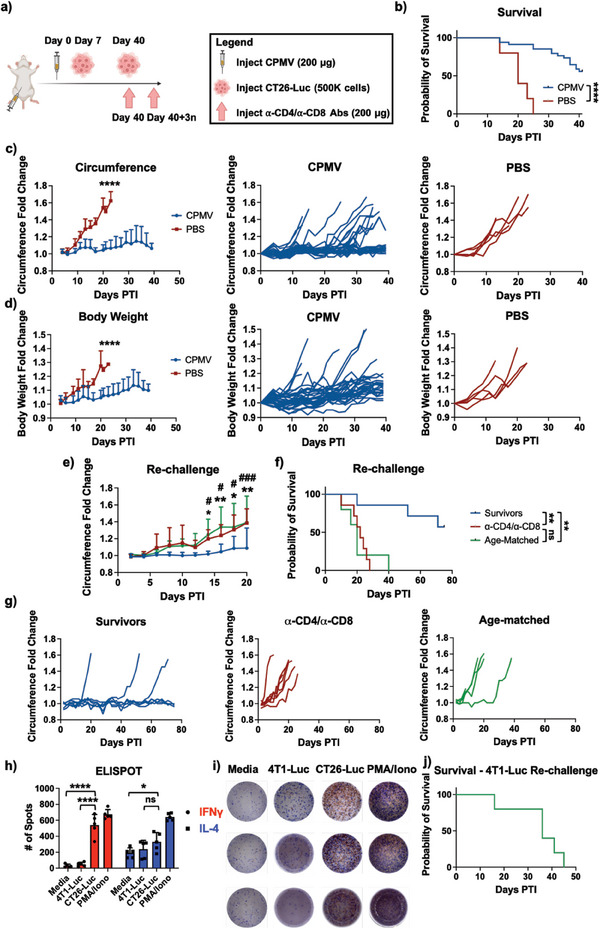
CPMV prophylaxis protects against CT26‐Luc metastasis. a) Injection schedule. BALB/C mice were given a single dose adjuvant therapy of CPMV (*n* = 35) followed by inoculation of 500000 CT26‐Luc cells i.p. Survivors were then re‐challenged at 40 days, and some of the survivors were also injected every 3 days with α‐CD4 and α‐CD8 NAbs. b) Survival of mice following CT26‐Luc challenge. *n* = 30 for CPMV and *n* = 5 for PBS. c) Average circumference fold change following CT26‐Luc challenge (left) and individual circumference fold change of CPMV (middle) and PBS (right) mice. *n* = 30 for CPMV and *n* = 5 for PBS d) Average body weight fold change following CT26‐Luc challenge (left) and individual body weight fold change curves of CPMV (middle) and PBS (right) mice. *n* = 30 for CPMV and *n* = 5 for PBS e) Average circumference fold change following re‐challenge. Age‐matched mice, which were not pre‐exposed to either CPMV or CT26‐Luc were used as the negative control. The *s represent the p‐value comparisons between the survivors and the age‐matched mice while the #s represent the p‐value comparisons between the survivors and the α‐CD4/α‐CD8 NAbs‐injected mice (*n* = 5–7). f) Survival of mice following re‐challenge. g) Individual circumference fold change graphs of the survivors (left), α‐CD4/α‐CD8 NAb‐injected mice (middle), and age‐matched controls (right). h,i) ELISPOT data from splenocytes of survivors following initial challenge (*n* = 3). h) The splenocytes were stimulated with media, 4T1‐Luc (negative control), CT26‐Luc, and PMA/iono (positive control), and the number of spots were plotted. i) Qualitative analysis of individual wells from the ELISPOT. j) Survival curve of mice re‐challenged with 4T1‐Luc s.c. */# = *p* < 0.05, ** = *p* < 0.01, ### = *p* < 0.001, **** = *p* < 0.0001, ns = not significant. The values displayed are all average values while the error bars represent the standard deviations. Graphs c–e) were analyzed using two‐way ANOVA, b) and f) were analyzed with a Log‐rank (Mantel‐Cox) test, and h) was analyzed with Student's T‐test. The schematic in a) was created on Biorender.com.

Metastatic cancers in the intraperitoneal (i.p.) space have poor prognoses and when at advanced stages are one of the deadliest forms of cancer.^[^ ^25^, ^26^, ^27^ ^]^ Therefore, as an initial study, we investigated whether CPMV could prevent settling and dissemination of i.p. metastases from i.p. challenge using CT26‐Luc colon cancer cells (**Figure** **2**). CPMV was administered into the i.p. space (200 µg), and mice were challenged after 1 week with CT26‐Luc tumors (500000 cells, i.p.) (Figure 2a). Mice that received CPMV treatment demonstrated significantly extended survival compared to the PBS control mice with 56% of mice surviving the initial tumor challenge while all control mice succumbed to the disease within 25 days (Figure 2b). The growth of the CT26‐Luc within the i.p. space can be followed by measuring the abdominal circumference and body weight of the mice, which increase following ascites development and enlargement of the mouse peritoneal cavity. By day 20 post tumor injection (PTI), the average circumference and body weight of the mice in the CPMV group increased by only 4.4 and 4.6%, respectively, while in the PBS group the circumference and body weight had increased to 54.2 and 27.4%, respectively (Figure 2c,d) corresponding to a comparative increase of 12.3 and 6‐fold compared to treated mice.

**Figure 3:**
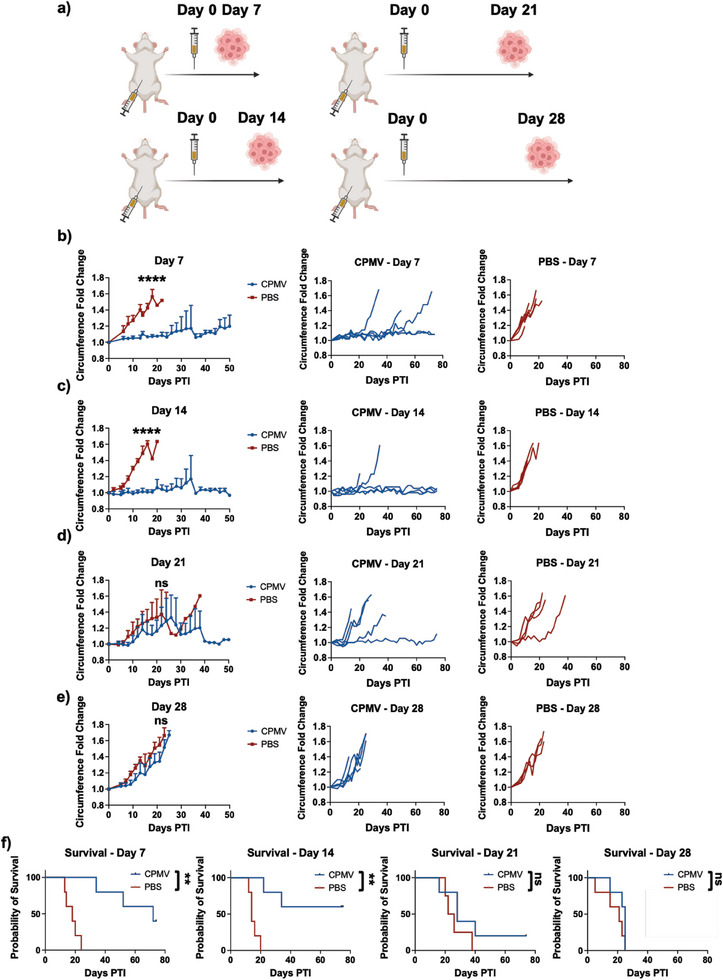
Longitudinal analysis of CPMV mediated CT26‐Luc protection. a) Injection schedule. CPMV was injected i.p. in BALB/C mice followed by injection of CT26‐Luc cells i.p. after 7, 14, 21, and 28 days. b–e) Average circumference fold change of mice with b) 7, c) 14, d) 21, and e) 28 days between CPMV and CT26‐Luc injection (left), and the individual circumference fold change curves for CPMV (middle) and PBS (right). f) Survival curves of mice with 7 (left), 14 (middle left), 21 (middle right), and 28 (right) days between the CPMV and CT26‐Luc injection. Curves b‐e) were analyzed by two‐way ANOVA and are displaying the average circumference fold change with the error bars representing the standard deviation. Graph f) was analyzed using a log‐rank (Mantel‐Cox) test. ** = *p* < 0.01, **** = *p* < 0.0001, ns = not significant. All experiments were done with *n* = 5. The schematic in a) was created on Biorender.com.

**Figure 4:**
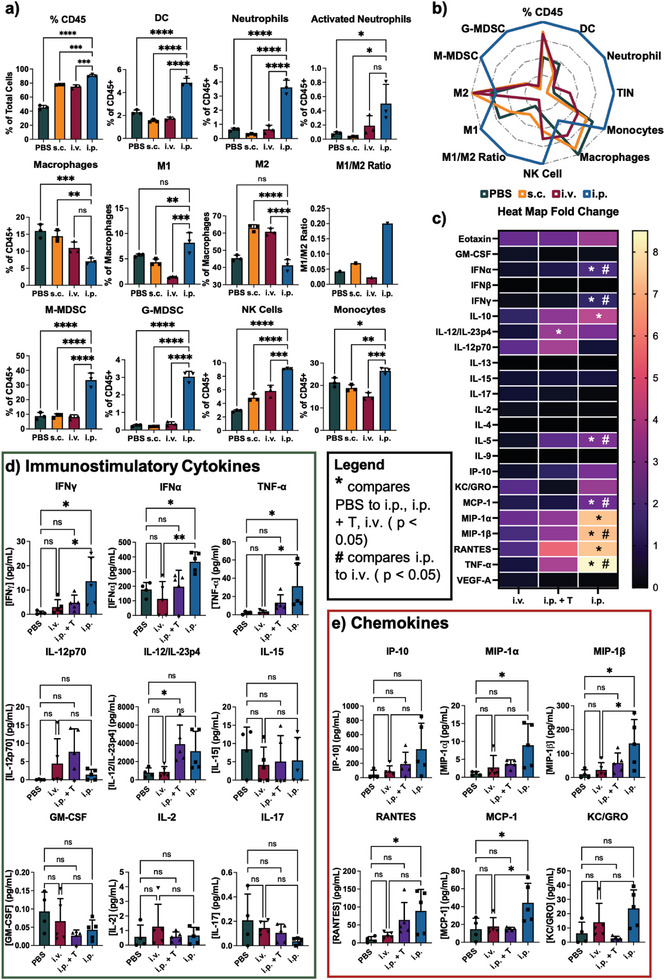
Mechanism of action of CPMV prophylaxis. a) Flow cytometry data of i.p. fluid following PBS, CPMV i.v., CPMV s.c., and CPMV i.p. injection. The samples were read in triplicate from the combined i.p. fluid of *n* = 3 mice. b) Spider plot of the data in Figure 4a. The groups were plotted as fold‐changes against the group that had the highest level of that specific cell. c) Heat map showcasing the fold change in expression of the MSD data in d) and e) compared to PBS. * is comparing any of the i.p., i.p. + T, or i.v. data to PBS (*p* < 0.05) while the # is comparing the i.p. to i.v. (*p* < 0.05). d) MSD data of a select group of immunostimulatory cytokines (*n* = 4–5). e) MSD data of chemokines (*n* = 4–5). The data in a), d), and e) represent the average values, and the errors bars are showing the standard deviation. The groups were compared using one way‐ANOVA. The flow data was run in triplicate while the MSD was run at *n* = 4–5. * = *p* < 0.05, ** = *p* < 0.01, *** = *p* < 0.001, **** = *p* < 0.0001, ns = not significant. The MSD analysis of additionally investigated cytokines can be found in Figure S9 (Supporting Information).

**Figure 5:**
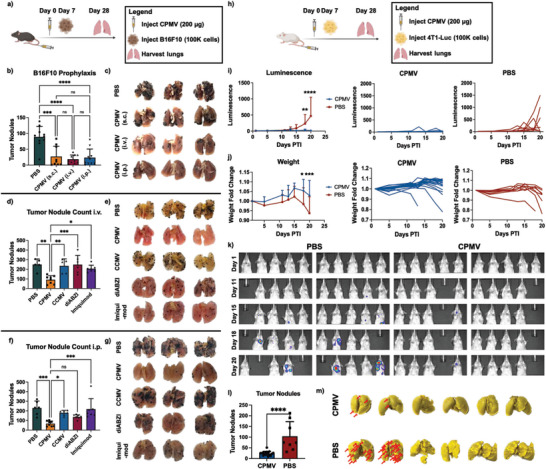
CPMV reduces lung B16F10 and 4T1‐Luc tumor seeding prophylactically. a) Injection schedule for B16F10. CPMV is injected i.v. prophylactically in C57BL/6J mice (*n* = 6–10) followed by injection of B16F10 cells i.v. after one week. The lungs are harvested 3 weeks PTI. b) Quantitative analysis of counted tumor nodules following lung harvesting. CPMV adjuvant therapy is administered s.c., i.v., or i.p. while PBS was injected i.v. (*n* = 6–10) c) Qualitative imaging of lungs from b) following lung harvesting. The lungs are fixed and then manually counted after harvesting. d) CPMV prophylaxis is compared to other adjuvants such as CCMV, diABZI, and imiquimod when injected i.v. CCMV is a similar plant virus to CPMV, diABZI is a STING agonist, and imiquimod is a TLR7 agonist. (*n* = 5–8) e) Qualitative imaging of lungs from d) following lung harvesting. f) Quantitative analysis of tumor nodules comparing CPMV to CCMV, diABZI, and imiquimod when injected i.p. (*n* = 6–8) g) Qualitative imaging of lungs from f) following lung harvesting. h) Injection schedule for 4T1‐Luc. CPMV is injected in BALB/C mice (*n* = 9–10) followed by injection of 4T1‐Luc i.v. after one week. The lungs are harvested 3 weeks after initial i.v. injection. i) Luminescent outgrowths of the 4T1‐Luc within the lungs as measured by IVIS. The figure is showing average luminescence (left), individual curves for CPMV (middle), and PBS (right). j) Weight fold change in mice with respect to time. The figure is showing average weight fold change (left), individual curves for CPMV (middle), and individual curves for PBS (right). k) IVIS images of the mice. The colored areas represent areas of tumor development. l) Tumor nodule counting. The tumor nodules were manually counted as with the B16F10 mice following Bouin staining. m) Qualitative images of the lungs counted in l). The red arrows are pointing to the areas of tumor nodule development. The schematics in a) and h) were created on Biorender.com. The lung tumor nodules for the B16F10 were analyzed using one‐way ANOVA, and the 4T1‐Luc were analyzed using Student's T‐Test. The luminescence and weight measurements were analyzed using two‐way ANOVA. The data in b), d), f), i), j), and l) represent the average values, and the error bars are showing the standard deviations. * = *p* < 0.05, ** = *p* < 0.01, *** = *p* < 0.001, **** = *p* < 0.0001, and ns = not significant. The lungs in c), e), g), and m) are representative images taken and are not the only lungs analyzed.

At day 40, the surviving mice were re‐challenged with CT26‐Luc (500000 cells, i.p.) (Figure 2a) – 5 of the mice were not re‐challenged and used for ELISPOT analysis (see below). T cell depletion studies were simultaneously carried out, and T cells were depleted using anti‐CD4 (α‐CD4) and anti‐CD8α (α‐CD8) neutralizing antibodies (NAbs) every 3 days to elucidate the importance of the T cell response following re‐challenge. Age‐matched mice with no prior CT26‐Luc exposure served as a control; age‐matching was utilized to ensure that age does not play a factor in the response against the re‐challenge. Similar to the initial challenge, the circumference increased quickly in the control mice (Figure 2e,g); in comparison, the tumors in the survivors of the initial challenge (from here on out referred to as the “survivors”) showed markedly diminished tumor growth. The importance of the T cell response was also demonstrated, as the tumors in the NAb‐administered mice grew at similar rates as with the age‐matched controls. By day 20 following the re‐challenge, the circumference for the survivors, NAb‐injected mice, and age‐matched mice were 8.9, 38.4, and 39.2% greater than before the re‐challenge, respectively. Survival following re‐challenge was also markedly improved in the survivor group compared to controls (Figure 2f). A median survival in the survivor's group could not be determined due to there being >50% survival while in the NAb‐injected mice and the age‐matched controls, the median survival was 22 and 20 days, respectively. The weight of the mice following re‐challenge is found in Figure S1 (Supporting Information). This data indicates that boosting the immune system with CPMV prevents onset of metastases in the i.p. space and primes long‐lasting T cell‐dependent antitumor immunity.

To corroborate the importance of the T cell response as well as demonstrate the formation of immune memory, splenocytes from 5 survivors were harvested and subjected to an enzyme‐linked immunosorbent spot (ELISpot) assay (Figure 2h,i). The splenocytes were isolated and stimulated with media, 4T1‐Luc triple negative breast cancer (TNBC) (negative control), CT26‐Luc, and phorbol 12‐myristate 13‐acetate (PMA)/ionomycin (iono) (positive control). The number of red spots, which measures IFNγ production, produced by the CT26‐Luc‐stimulated splenocytes was significantly higher than in the media or 4T1‐Luc signifying a potent cytotoxic T lymphocyte (CTL)/Th1 response which was tumor specific. The CT26‐Luc stimulation produced 539.8 spots, while the media and 4T1‐Luc produced 31.4 and 48.2 spots, respectively. IL‐4 production, measured by blue spots and which signifies a Th2 response, was significantly greater in the CT26‐Luc samples when compared to media. The specific response to CT26‐Luc stimulation demonstrates that following initial tumor killing, immune memory against the initial tumor is established – stimulation with a control 4T1‐Luc TNBC cell line induced no IFNγ production. As shown with the neutralization of CD4 and CD8 (Figure 2e–g), this T cell response seems to be vital. Indeed, when ELISpot was ran on splenocytes of mice that survived an initial challenge, but not a re‐challenge, T cell response against the CT26‐Luc was nonexistent (Figure S2, Supporting Information).

To demonstrate that the adaptive immune response elicited was indeed tumor‐specific, we performed another re‐challenge experiment comparing re‐challenge with CT26‐Luc (as described above) and 4T1‐Luc. The initial tumor prevention data were in good agreement with the Figure 2 demonstrating good reproducibility (Figure S3a–d, Supporting Information). The survivors were then split into 2 groups: one re‐challenged with CT26‐Luc i.p. and the other with 4T1‐Luc s.c. Re‐challenge with CT26‐Luc again led to long‐term survivors due to the formation of immune memory (Figure S3e, Supporting Information). However, in the mice injected with the 4T1‐Luc, the tumors grew unabated, and there were no survivors (Figure 2j; Figure S3f, Supporting Information). To demonstrate that the immune memory is systemic and tissue agnostic, the experiment was repeated and surviving mice were re‐challenged with 100000 CT26‐Luc cells s.c. away from the original injection site which was i.p. (Figure S3g, Supporting Information). By day 50 PTI, 9/10 of the survivors remained alive with complete tumor rejection while 5/7 of the mice in the age‐matched mice had succumbed to the CT26‐Luc (Figure S3h, Supporting Information). At day 27 PTI, the average tumor volume was 0 mm^3^ for the survivors and 639.4 mm^3^ for the age‐matched mice (Figure S3i, Supporting Information). Overall, the re‐challenge experiments demonstrate that CPMV prophylaxis generates potent immune memory after initial challenge that is tumor‐specific, but tissue‐agnostic.

Lastly, patients oftentimes are diagnosed with i.p. metastases already present, as current imaging techniques are not well suited for early diagnosis and screening of i.p. tumor nodules.^[^ ^25^ ^]^ Therefore, to better mirror a clinical situation, mice were challenged with a low number of CT26‐Luc cells (50000 cells, i.p.) prior to CPMV prophylactic treatment, which was then followed by CT26‐Luc challenge as described above (Figure S4a, Supporting Information). In this scenario, CPMV adjuvant therapy remained effective even with metastatic cells already present within the i.p. space (Figure S4b–d, Supporting Information). Compared to PBS, CPMV increased the median survival from 19 to 39 days (Figure S4b, Supporting Information), and decreased circumference by 3.7‐fold 19 days PTI (Figure S4c, Supporting Information).

Contemporary treatment strategies, such as systemically administered chemotherapeutics and even localized i.p. chemotherapeutics are largely ineffective.^[^ ^28^, ^29^, ^30^ ^]^ Furthermore, peritoneal metastases are difficult to diagnose with common imaging techniques due to their small size and oftentimes go unnoticed.^[^ ^25^ ^]^ Therefore, a prophylactic approach such as the one proposed with CPMV that immediately prevents such outgrowths could be vital in the treatment of these cancers.

### 2.3 Longitudinal Analysis of CPMV Prophylaxis Against CT26‐Luc Colon Cancer

To test the duration of immune protection after CPMV prophylaxis, a longitudinal analysis of CT26‐Luc was carried out with the tumors injected after 7, 14, 21, and 28 days following CPMV prophylactic treatment (**Figure** **3**a; Figure S5a, Supporting Information). CPMV treatment protected from tumor challenge 7 days post treatment as demonstrated in Figure 2 (Figure 3b; Figure S5b, Supporting Information). Impressively, after only a single injection, efficacy continued to remain steadfast after 14 days (Figure 3c; Figure S5c, Supporting Information). In the 14‐day mice, compared to the PBS, CPMV reduced the circumference and body weight by 96.5‐ and 10.4‐fold, respectively, 12 days PTI (Figure 3c; Figure S5c, Supporting Information). Additionally, CPMV extended survival with 3/5 mice showcasing long‐term survival up to 75 days PTI, which was when the experiment was concluded, while all PBS mice reached clinical endpoints by day 20 (Figure 3f).

However, in mice challenged with tumors at 21‐ or 28‐days post CPMV treatment, the efficacy of the CPMV prophylaxis was completely abolished (Figure 3d–f; Figure S5d,e, Supporting Information), and there were no significant differences in the circumference and body weight changes between groups. One mouse in the CPMV and 21‐day tumor challenge group did survive long‐term, which was considered statistically insignificant (Figure 3f).

Regardless, CPMV was able to significantly delay tumor growth and extend survival when tumor challenge was carried within a 14‐day window, indicating durable effects of the CPMV prophylactic treatment. The persistence of CPMV may be attributed to its nanoparticle character providing advantages over small molecules therapeutics, which may suffer from rapid clearance.^[^ ^31^ ^]^ Additionally, CPMV is rather stable, which may aid its long‐lasting efficacy. CPMV has been shown to be stable in a wide range of pH values (mirroring the pH of the gastrointestinal (GI) tract) and temperatures thus demonstrating efficacy after a single injection.^[^ ^32^, ^33^ ^]^ The biodistribution of CPMV was investigated 7 and 14‐days following CPMV i.p. injection, which demonstrated that indeed CPMV remained detectable within the i.p. space within the 14‐day window (Figure S6, Supporting Information). Future studies could elucidate whether tumor protection could be extended further following multiple injections of CPMV.

### 2.4 Route of Administration of CPMV Affects Efficacy Against i.p. Metastases

We then further investigated whether CPMV could be administered through different routes and still provide broad protection against CT26‐Luc i.p. challenge. CPMV was injected either subcutaneously (s.c.), intravenously (i.v.), or i.p., and mice were challenged with CT26‐Luc cells i.p. as before. While i.p. injection of CPMV demonstrated significant protection, s.c. and i.v. injection of CPMV did not slow tumor growth or extend survival (Figure S7, Supporting Information). There were no significant differences between PBS and mice injected with CPMV i.v. or s.c., which demonstrated that for i.p. prophylaxis, the CPMV route of administration has to be localized to the area of tumor development.

To probe the mechanism of action and to gain understanding why i.p. but not i.v. or s.c. administration elicited protection against tumor challenge, flow cytometry and cytokine multiplexed analyses were carried out using i.p. fluid collected 1 week post CPMV injection. While i.p. administration of CPMV significantly improved the recruitment of antitumor immune cells in the i.p. space (**Figure** **4**a,b), this was not observed when CPMV was administered i.v. or s.c. Compared to controls, i.p. administration of CPMV increased the population of CD45+ cells within the i.p. space; specifically dendritic cells (DCs), neutrophils, activated neutrophils, monocytes, and natural killer (NK) cells were increased. Surprisingly, the percentage of macrophages was significantly decreased following i.p. treatment with CPMV, but when dissecting the phenotypic makeup of these macrophages, the i.p. injection of CPMV led to the polarization/recruitment of M1 macrophages and reduction of M2 macrophages thus displaying a greater M1/M2 ratio – this was in stark contrast to the mice receiving PBS or CPMV i.v. or s.c. Tumor‐associated macrophages (TAMs) are one of the major causes of immunosuppression within the TME leading to T cell exhaustion, release of immunosuppressive cytokines, and increase in metastasis^[^ ^34^ ^]^ – therefore, decreasing the overall macrophage population while polarizing the remaining macrophages to antitumor phenotypes may be a key factor toward the successful immunoprevention of tumor growth in mice receiving the i.p. CPMV treatment.

It must also be noted that we did observe increases in both the monocytic and granulocytic myeloid‐derived suppressor cell (M/G‐MDSC) populations. This may be a byproduct of the CPMV immune cascade – previous studies have demonstrated that CPMV causes significant release of IL‐6, which has both immunostimulatory and immunosuppressive effects.^[^ ^17^, ^35^ ^]^ While IL‐6 can improve the T cell response, it also causes the recruitment of MDSC populations.^[^ ^35^, ^36^ ^]^ Regardless, the increase in the G/M‐MDSC populations do not seem to affect the potency of CPMV prophylaxis, and MDSCs can also be polarized into antitumor phenotypes. In summary, the injections of CPMV i.v. and s.c. did not induce immune cell recruitment into the i.p. space compared to CPMV i.p. injection – this aligns with the efficacy data (Figure S7, Supporting Information) in that CPMV i.p. and not i.v. or s.c. offers protection against CT26‐Luc challenge. The gating strategy can be found in Figure S8 (Supporting Information).

To probe the cytokine/chemokine response, MSD U‐PLEX assay was carried out on the i.p. fluid one week after CPMV i.p. and i.v. injection (Figure 4c–e; Figure S9, Supporting Information). A second study was done where CPMV was administered i.p. followed by i.p. CT26‐Luc tumor challenge 1 week post CPMV therapy; i.p. fluid was then collected 24 h PTI (this sample is labeled i.p. + T). Similar to previous Luminex data,^[^ ^20^ ^]^ CPMV was able to induce upregulation of immunostimulatory cytokines notably IFNγ, interferon α (IFNα), TNF‐α, interleukin‐12 (IL‐12p70), and IL‐12/IL‐23p4. While non‐infectious toward mammals, CPMV is recognized by PRRs and stimulates an anti‐viral response inducing IFN expression.^[^ ^37^ ^]^ Although not significant, we did notice trends toward increased IL‐12p70 and IL‐12/IL‐23p4 when tumor challenge was performed. Additionally, chemokines were significantly upregulated following CPMV i.p. injection (Figure 4e). Compared to PBS, CPMV increased the production of interferon gamma‐induced protein 10 (IP‐10), macrophage inflammatory protein‐1α (MIP‐1α), MIP‐1β, regulated on activation, normal T cell expressed and secreted (RANTES), monocyte chemoattractant protein‐1 (MCP‐1), and keratinocyte chemoattractant (KC)/human growth‐regulated oncogene (GRO) by 9.4‐, 9‐, 10.6‐, 9.8‐, 3‐, and 3.8‐fold. These chemokines act as chemoattractants for a wide range of both innate and adaptive immune cells, which mirrors the finding of increased immune cell infiltration in the i.p. space upon CPMV treatment (Figure 4a,b). For instance, MCP‐1 has been demonstrated to induce monocyte and DC recruitment, and the flow data corroborates that only CPMV i.p. increases both the monocyte and DC populations. The i.v. CPMV dose does not induce expression of cytokines or chemokines (Figure 4c–e), consistent with lack of immune cell recruitment (Figure 4a) and lack of efficacy (Figure S7, Supporting Information).

Proteomic studies using tandem mass tag (TMT) were also performed; significant changes were denoted as any protein with a significance value above 15 and an expression ratio either < 0.5 or >2.28 proteins were upregulated within the i.p. space following CPMV injection, and 5 were downregulated (Figure S10, Supporting Information). Of note, IgG2a and IgG2b were one of the most upregulated proteins after CPMV injection – both IgG subtypes are vital in viral clearance.^[^ ^38^ ^]^ However, TMT does not provide any information on the antigen specificity of the IgG2 although it is understood that i.p. administration of CPMV leads to generation of α‐CPMV antibodies.^[^ ^39^ ^]^ Others have demonstrated that injection of tumor‐independent IgG can induce tumor regression in both prophylactic and treatment settings.^[^ ^40^, ^41^, ^42^ ^]^ Therefore, it may be that the increase in IgGs is contributing to tumor rejection although this has not been experimentally validated. Indeed, others show that supernatant from IgG‐stimulated macrophages can induce considerable tumor cell death.^[^ ^43^ ^]^ CPMV injection also led to increased production of fibronectin and peroxiredoxin‐1, which have been shown to improve chemotaxis and leukocyte migration and induces IFN production/aids in the antiviral activity of CD8+ T cells.^[^ ^44^, ^45^, ^46^, ^47^ ^]^ Lastly, proteins related to amino acid and lipid metabolism (e.g., betaine‐homocysteine S‐methyltransferase and fatty acid‐binding protein) as well as ATP‐generating pathways such as glycolysis (e.g., S‐adenosylmethionine synthase isoform type‐1 and fructose‐biphosphate aldolase B) were also increased.

Overall, CPMV led to significant increases in cyto/chemokines and immune cells into the i.p. space. While this may not directly lead to tumor killing, we hypothesize that the recruited immune cells can home at an improved rate into the tumor lesions found within the peritoneal cavity due to their now proximity to the metastatic tumors. Additionally, these cells are better geared for tumor killing due to the immunostimulatory nature of the i.p. microenvironment as seen by the increased levels of activated neutrophils and the M1/M2 ratio (Figure 4a). Lastly, the recruited immune cells release additional immunostimulatory cytokines such as IFNγ and TNFα that can have direct and indirect antitumor effects.

### 2.5 CPMV Prophylaxis is Applicable Across Tumor Types

To demonstrate broad applicability of the CPMV prophylactic treatment, we also tested efficacy of i.p. administered CPMV against i.p. challenge with ID8‐*Defb29/Vegf‐A* cells, a model of ovarian cancer (Figure S11a, Supporting Information). Again, mice receiving the CPMV adjuvant therapy demonstrated significantly improved survival rates with a median survival of 52 for the CPMV group versus 45 days for the control group (Figure S11b, Supporting Information). There were also long‐term survivors for the CPMV group, which were not seen in the PBS‐injected mice. With CPMV, the circumference and weight of the mice 45 days PTI increased an average of 22 and 20.1%, respectively (Figure S11c,d, Supporting Information). However, in the PBS mice, the circumference and weight increased 62.2 and 42.6%, a comparative 2.8‐ and 2.1‐fold increase compared to CPMV, respectively. The survivors in the CPMV group were analyzed via ELISpot on day 75 PTI (Figure S11e,f, Supporting Information), which indicated that the splenocytes from the surviving mice were able to produce IFNγ following ID8‐*Defb29/Vegf‐A* stimulation indicating immune memory and a Th1‐dependent T cell response against the original tumor. On the other hand, stimulation with a control B16F10 cell line did not produce any IFNγ spots. Representative images of the ELISpot can be found in Figure S11f (Supporting Information).

### 2.6 B16F10 Melanoma Lung Metastasis Prophylaxis with CPMV

Another major hurdle in the treatment of cancer is the metastasis of cancer cells to the lungs.^[^ ^48^ ^]^ In fact, lung metastases are extremely common, and can be found in a myriad of cancer types ranging from breast, colorectal, uterine and melanoma.^[^ ^49^ ^]^ Prognosis is poor, and survival rates are low for lung metastatic tumors; systemically administered therapeutics are oftentimes ineffective due to dose‐limiting toxicity and drug resistance.^[^ ^50^, ^51^, ^52^ ^]^ Therefore, we evaluated efficacy of CPMV as a prophylactic measure against B16F10 melanoma lung metastases. CPMV (200 µg) was administered s.c., i.v., and i.p., and tumor challenge was performed one week post treatment using B16F10 melanoma cells (100000 cells, injected i.v.) (**Figure** **5**a). The lungs were harvested 3 weeks PTI, and the tumor nodules were manually counted. Interestingly, unlike with the i.p. prophylaxis, the route of administration was insignificant, and all administration routes considerably depreciated the number of metastatic tumor nodules in the lung (Figure 5b,c). Compared to PBS, there was a noticeable 3.3‐, 3.8‐, and 4.8‐fold decrease in the number of tumor nodules following s.c., i.p., and i.v. injection, respectively. Qualitatively, the difference is also appreciable as seen in Figure 5c.

CPMV is unique in its ability to instigate an immune response – compared to other plant viruses, bacteriophages, and some mammalian viruses, it performs significantly better as a cancer immunotherapy.^[^ ^24^ ^]^ Therefore, the ability of CPMV was compared head‐to‐head against other adjuvants to see whether the prophylactic ability was unique to CPMV (Figure 5d–g). diABZI is a stimulator of interferon genes (STING) agonist while imiquimod is a TLR7 agonist.^[^ ^53^, ^54^ ^]^ As a negative control, the cowpea chlorotic mottle virus (CCMV), which has the same size and shape, is negatively charged, and is grown in the same plants as CPMV but is non‐immunomodulatory in the context of cancer immunotherapy,^[^ ^55^ ^]^ was utilized. The reagents were compared in both an i.p. and i.v. setting, and with both injection routes, CPMV fared better in reducing tumor nodules compared to any other adjuvant tested. When injected i.v., CPMV decreased tumor nodule counts compared to PBS, CCMV, diABZI, and imiquimod by 2.7‐, 2.6‐, 2.7‐, and 2.2‐fold, respectively (Figure 5d,e). When injected i.p., CPMV improved tumor nodule rejection with 3.4‐, 2.7‐, 2‐, and 3.2‐fold reductions compared to PBS, CCMV, diABZI, and imiquimod, respectively (Figure 5f,g). Unlike diABZI and imiquimod, which are small molecule agonists, CPMV may not suffer from immediate washout and rapid degradation thereby prolonging its immunostimulatory effects. Additionally, diABZI and imiquimod display significant immunotoxicity as systemically administered agents while CPMV has been injected at doses up to 100 mg kg^−1^ with no observed toxicities.^[^ ^56^ ^]^ In the clinic, one might expect that longer therapeutic windows such as with CPMV may be preferred to prolong protection against metastatic outgrowths with the least number of doses.

The immunostimulatory nature of the reagents was also compared using the RAW264.7‐Luc NFκB reporter line. Data are in agreement and support the in vivo study; CPMV significantly outperformed the other reagents in the stimulation of the RAW264.7 macrophages (Figure S12, Supporting Information). The luminescent output of CPMV was 566.7 compared to 338, 399.7, 432, and 394.7 of media, CCMV, diABZi, and imiquimod, respectively. Therefore, while the other reagents could induce NFκB signaling compared to PBS, it was significantly lower than that of CPMV. The triple‐pronged TLR activation of CPMV along with its multivalent nature likely leads to avidity effects and enhanced presentation of T helper epitopes;^[^ ^57^ ^]^ the nanoparticle character may explain the unmatched efficacy of CPMV compared to other adjuvants.

### 2.7 CPMV Prophylaxis Efficacy in a 4T1‐Luc TNBC Lung Metastatic Tumor Model

To verify that CPMV is efficacious in a different tumor model, a 4T1‐Luc TNBC cell line was utilized in BALB/C mice. Following i.v. treatment using CPMV, mice were challenged with 4T1‐Luc cells (100000 cells, i.v.) Disease burden was followed by luminescent imaging every 2 days. After 3 weeks, the lungs were harvested and the tumor nodules were manually counted (Figure 5h). In the CPMV mice, the lungs displayed little to no tumor development, while in the PBS mice, the luminescence increased rapidly (Figure 5i) indicating tumor development, which led to weight loss (Figure 5j). By day 20 PTI, the luminescent counts within the PBS mice had reached 468.3 while the CPMV mice were 18.2, a 25.7‐fold decrease. Representative imaging of mice is found in Figure 5k. Manual counting of the tumor nodules from the harvested lungs also demonstrated that CPMV adjuvant therapy decreased the average number of nodules from 104 to 24, a 4.3‐fold decrease (Figure 5l,m). Even qualitatively, the differences are pronounced as can be seen by the red arrows in the first two lungs, which are pointing to areas of tumor growth (Figure 5m).

### 2.8 CPMV Elicits Significant Systemic Immunostimulation Causing the Reduction of Tumor Nodules

**Figure 6:**
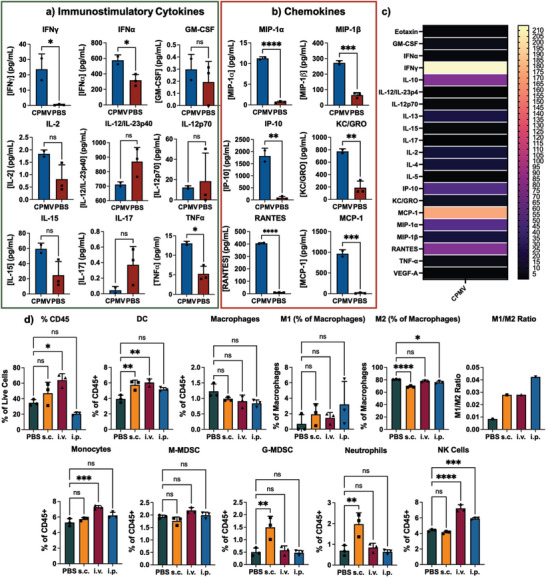
Mechanism of action of CPMV immunoprophylaxis. a) MSD data of immunostimulatory cytokines following i.v. injection of CPMV or PBS. b) MSD data of chemokines following i.v. injection of CPMV or PBS. c) Heat map showcasing the fold change of cytokines/chemokines in a) and b) comparing CPMV i.v. to PBS. MSD data of other cytokines can be found in Figure S13 (Supporting Information). d) Flow cytometry data of the blood of mice following CPMV s.c., i.v., and i.p. injection. The MSD data was analyzed using Student's T‐test (*n* = 2–3), and the flow cytometry was analyzed using one‐way ANOVA and run in triplicate from a combined pool of blood from *n* = 3 mice. The values represent the average values, and error bars are representing the standard deviation. * = *p* < 0.05, ** = *p* < 0.01, *** = *p* < 0.001, **** = *p* < 0.0001, ns = not significant.

The mechanism of action of the CPMV‐induced reduction of tumor nodules was further characterized by MSD analysis of blood samples collected 1‐day after i.v. CPMV treatment (**Figure** **6**a–c; Figure S13, Supporting Information). CPMV administration led to significant increases in immunostimulatory cytokines and chemokines within the plasma of the mice. Notably, there were significant increases in IFNγ, IFNα, and TNF‐α with 48.1‐, 1.8‐, and 2.5‐fold increases, respectively. With the chemokines, CPMV injection led to significant increases in all tested chemokines (Figure 6b). A heat map showing the fold‐changes of the cytokines/chemokines following CPMV i.v. injection compared to PBS is shown in Figure 6c. Overall this data was comparable to the observations made after i.p. administration (see Figure 4).

Next, changes in immune cell populations within the blood were assayed; here, we considered all routes of administration: s.c., i.v., and i.p. (Figure 6d). While only i.p. administration leads to an increase of immune cells within the i.p. space (Figure 4), trends in immune profiles within the blood are distinct, but overall increased independent of route of administration. For instance, neutrophil populations were increased significantly following s.c. injection, but not i.p. or i.v. On the other hand, nature killer (NK) cells were only increased by i.p. and i.v. injection, but not s.c. Notably, the M1/M2 ratio is increased independent of injection route. Overall, the panels indicate significant activation of immune cells in blood; in stark contrast, flow cytometry of the spleens or lungs of the mice only showcased minimal differences in immune cell populations (Figure S14, Supporting Information). This data suggests that CPMV may assert efficacy through the clearance and processing of circulating tumor cells (CTCs) within the blood. The data also mirrors the metastatic tumor prophylaxis data (Figure 5d–g) in that CPMV is effective regardless of administration route. However, for different indications, different administration routes may be more effective, and future studies should probe how the differences that are seen in the recruited cell type could affect tumor clearance.

### 2.9 Surgical Removal of Primary Tumors Followed by CPMV Adjuvant Therapy

**Figure 7:**
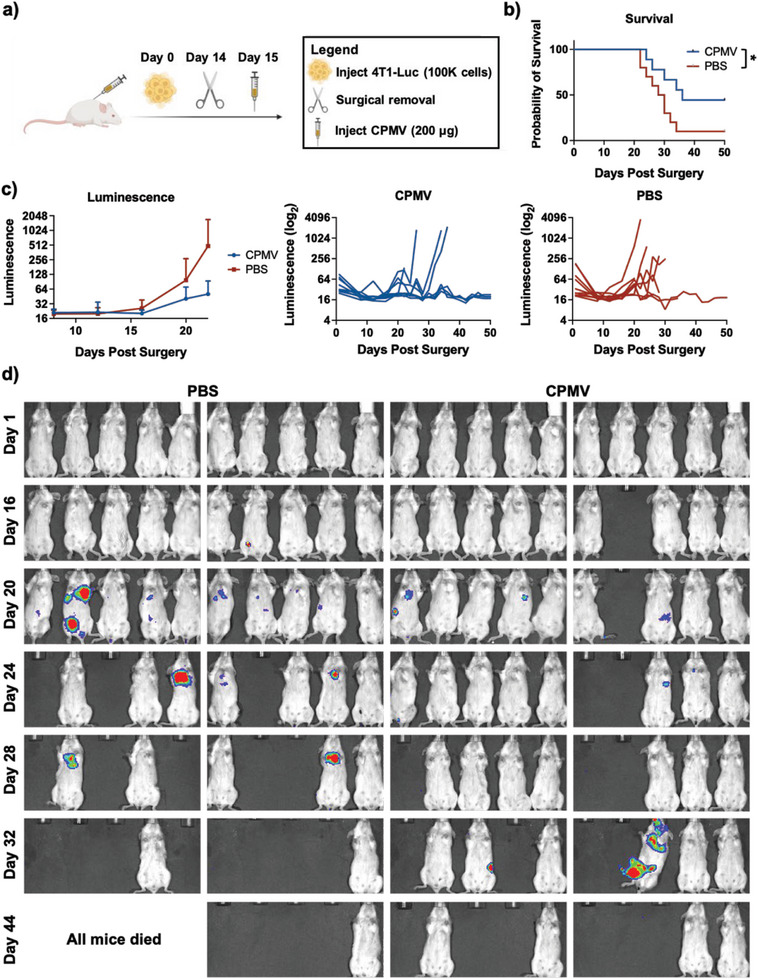
4T1‐Luc primary surgery study combined with CPMV adjuvant therapy. a) Injection schedule schematic. Mice were injected with CPMV i.v. one day after surgical removal of a primary TNBC tumor (*n* = 10). b) Survival curve of mice given either CPMV or PBS adjuvant therapy. c) Luminescence measured by IVIS of mice. The figure is showing the average luminescence (left), individual luminescent curves for CPMV (middle), and individual luminescent curves for PBS (right). The values represent the average values, and the error bars represent the standard deviation. d) Representative IVIS images of the mice. The colored areas represent areas of tumor growth. The survival was analyzed using a log‐rank (Mantel‐Cox) test. * = *p* < 0.05. The graphic in a) was created on Biorender.com.

To test the efficacy of CPMV in a more clinically relevant model, a surgery study was performed. Female BALB/C mice were inoculated with 4T1‐Luc (100000 cells, s.c.) followed by surgical removal of the tumor after 2 weeks (**Figure** **7**a). The following day, the mice were injected i.v. with CPMV as adjuvant therapy, and mice were monitored by IVIS for signs of metastasis and recurrence. Compared to PBS mice, CPMV administration led to a significant improvement in survival with a median survival of 36 days for CPMV and 29 days for PBS (Figure 7b). CPMV prophylaxis also improved the number of long‐term survivors by 400%. The luminescence of the 4T1‐Luc tumors was measured, which demonstrated that the adjuvant therapy decreased the growth of the tumors albeit insignificantly (Figure 7c). The luminescent imaging of the mice can be found in Figure 7d, which highlights that CPMV adjuvant therapy decreased rates of tumor growth both in terms of recurrence and metastasis.

Together, data indicate that the CPMV prophylactic adjuvant therapy has broad efficacy and is mouse strain independent (C57BL/6J and BALB/C mice), applicable to various tumor types (colon, ovarian, melanoma, and TNBC), tumor site independent (i.p. metastases, lung metastases, and s.c. tumors), able to generate systemic immune memory (both i.p. and s.c. re‐challenge and confirmed through ELISpot) and clinically relevant (pre‐disseminated i.p. disease, surgical removal of primary tumors).

## 3 Conclusion

Metastatic cancers contribute the highest percentage of cancer‐related deaths, and current treatment options for metastatic cancers remain limited. Adjuvant therapy has greatly improved outcomes in breast and colon cancer, and immunotherapies may play a significant role in future adjuvant treatment. CPMV is an immunostimulatory adjuvant that when injected intratumorally leads to recruitment of immunostimulatory cells, release of antitumor cytokines, and potent tumor cell death. We show here that CPMV also demonstrates significant utility in the adjuvant treatment and rejection of 4 metastatic models of murine cancer. When injected i.p. or i.v., CPMV causes the recruitment of a wide range of immune cells into either the i.p. space or blood, respectively, including DCs, neutrophils, and NK cells, which go on to release antitumor cytokines such as IFNγ, IFNα, TNF‐α, and IL‐12. This leads to the rejection of i.p. and i.v. metastases leading to long‐term survivors and building immune memory to protect against re‐challenge. The broad applicability and effectiveness of CPMV adjuvant therapy across a range of cancer types paired with the long‐lasting efficacy off of a single injection confers CPMV with the potential to be used as an adjuvant treatment in metastatic cancers.

## 4 Experimental Section

### Materials and Cells

Potassium phosphate monobasic, potassium phosphate dibasic anhydrous, GlutaMAX, HEPES, sodium pyruvate, Tween‐20, and 2‐mercaptoethanol were purchased from Fisher Scientific. Urea, Tris buffer, tris(2‐carboxyethyl)phosphine (TCEP), chloro‐acetamide, Lys C, trypsin, trifluoroacetic acid (TFA), acetonitrile (ACN), and formic acid were purchased from Thermo Fisher. PBS was purchased from G Biosciences. PMA was purchased from Millipore Sigma. Ionomycin, gadolinium(iii) chloride hexahydrate, and EDTA were purchased from Sigma‐Aldrich. Paraformaldehyde 32% (v/v) was purchased from Electron Microscopy Sciences. Fetal bovine serum (FBS) was purchased from R&D Systems. Ethanol (EtOH) was purchased from VWR. Penicillin/streptomycin (P/S) was purchased from Cytiva. RPMI‐1640 and DMEM were purchased from Corning.

CT26‐Luc and 4T1‐Luc cells were grown in RPMI‐1640 supplemented with 10% (v/v) FBS and 1% (v/v) P/S. B16F10 cells were grown in DMEM supplemented with 10% (v/v) FBS and 1% (v/v) P/S. ID8‐*Defb29/Vegf‐A* cells were grown in RPMI supplemented with 10% (v/v) FBS, 1% (v/v) P/S, 2 mm L‐glutamine, 1 mm sodium pyruvate, and 0.05 mm 2‐mercaptoethanol. RAW264.7‐Luc cells were thawed in DMEM supplemented with 10% (v/v) heat‐inactivated FBS, 1% (v/v) GlutaMAX (Gibco), and 1% P/S (Thaw Medium). After the first passage, the cells were moved to media consisting of the Thaw Medium plus 700 µg mL^−1^ of genecticin (Thermo Fisher) (Growth Medium 11A). All cells were grown at 37 °C and 5% CO_2_.

### CPMV purification and characterization

*UV–vis Spectroscopy*: CPMV was produced and purified in infected black eyed pea plants as done previously.^[^ ^58^ ^]^ UV–vis spectroscopy (Nanodrop) and Beer's law was used to measure the concentration: A = ε ∗ l ∗ c, where A = absorbance, ε = the extinction coefficient of CPMV (8.1 mL mg^−1^ cm^−1^), l = path length, and c = concentration. An A_260_/A_280_ value of ≈1.8 indicated pure particles.

*SDS‐PAGE*: CPMV (10 µg) was diluted in 1x lithium dodecyl sulfate sample buffer (Life Technologies) and 0.1 m KP, and heated for 5 min at 95 °C. The CPMV was loaded on to a 12% NuPAGE Gel (Thermo Fisher Scientific) and ran under the following conditions: 200 V, 120 mA, and 25 W in 1x MOPS buffer before imaging with an AlphaImager (Protein Simple).

*Agarose gel electrophoresis*: CPMV (10 µg) was diluted in 1x Gel Loading Purple Dye (Biolabs) and loaded into 0.8% (w/v) agarose gels with 1x GelRed nucleic acid gel stain (Gold Biotechnology). The samples were run at 120 V and 400 mA for 30 min.

*DLS*: CPMV was diluted to 0.1 mg mL^−1^ in DI water, and run at RT with a Zetasizer Nano ZSP/Zen5600 (Malvern Panalytical) using the Smolvchowski method.

*SEC*: SEC was carried out on a Superose 6 SEC column (column dimensions: 10 × 300 mm, exclusion limit 4 × 10^7^ M_r_, Cytiva) connected to an Äkta Explorer FPLC machine (Cytiva). Particle integrity was measured by the A_260_/A_280_ ratio at the elution peak.

*TEM*: Formvar coated carbon films (VWR International) were coated with 20 µL of 0.1 mg mL^−1^ CPMV diluted in DI water for 2 min. The CPMV solution was then replaced with 2% uranyl acetate for 2 min and allowed to dry before imaging on a Joel 1400 TEM.

### CPMV is Uniquely Immunogenic Compared to Other Viruses

The immunogenicity of CPMV was compared to the immunogenicity of other viruses using a RAW264.7‐Luc NFκB reporter line (RAW264.−7). The RAW264.7‐Luc cells were plated at a concentration of 30000 cells in 100 µL of Growth Media 11A, and 10 µg of CPMV, CCMV, Qβ, TMV, PhMV, and media were incubated for 48 h. CCMV and TMV were produced in black eyed peas and *Nicotiana benthamiana* plants, as done previously.^[^ ^55^, ^59^ ^]^ Qβ and PhMV were expressed in *E. coli* as done previously.^[^ ^60^, ^61^ ^]^ Luminescence was read using SteadyGlo Reagent (Promega) according to the manufacturer's instructions. The luminescent output was measured on a plate reader (Tecan).

### CPMV Adjuvant Therapy Against Metastatic CT26‐Luc Colon Cancer

All mice experiments were carried out in accordance with the guidelines set out by the Institutional Animal Care and Use Committee at the University of California San Diego, according to protocol S18021. All animals were purchased from the Jackson Laboratory and stored at the Moores Cancer Center and provided with unlimited food and drink.

6‐ to 8‐week‐old female BALB/C mice were purchased, and 200 µg of CPMV or PBS were injected intraperitoneally (i.p.). One week later, 500000 CT26‐Luc cells were injected i.p., and tumor growth was measured every 2 days by measuring the circumference and body weight of the mice. The circumference and body weight were used as indicators of tumor growth following i.p. tumor dissemination and ascites development. On day 40, the surviving mice were split into 2 groups and re‐challenged i.p. with 500000 CT26‐Luc cells. Naïve age‐matched mice were also challenged with 500000 CT26‐Luc cells. Mice were euthanized when the abdominal circumference increased >60% over the original circumference or when the body weight reached >75% over the original body weight or 30 g.

A separate group (*n* = 5) of the surviving mice following initial CT26‐Luc challenge were instead used for ELISPOT analysis. In brief, a 96‐well ELISPOT plate (Cellular Technology LTD) was coated with α‐mouse IFNγ and IL‐4 capture antibodies and stored overnight (ON) at 4 °C. The next day, the spleens of the mice were collected and digested into single‐cell suspensions using a spleen dissociation kit (Militenyi Biotec) according to the manufacturer's instructions. The splenocytes were then diluted to 500000 cells/100 µL in the CTL‐Test medium supplemented with 1% (v/v) GlutaMax. The cells were then stimulated with 100 µL of media only, 500000 4T1‐Luc cells, 50 ng mL^−1^ PMA and 1 µg mL^−1^ iono, or 500000 CT26‐Luc cells, and incubated at 37 °C and 5% CO_2_ for 48 h. The samples were then analyzed according to the manufacturer's instructions. The plate was imaged and the spots were quantified using an immunospot S6 ENTRY analyzer.

### Re‐Challenge Following CPMV Adjuvant Therapy

The re‐challenge experiment was repeated once more except the surviving mice were split into two groups and re‐challenged with 500000 CT26‐Luc cells i.p. or re‐challenged subcutaneously (s.c.) with 200000 4T1‐Luc cells. Tumors in the 4T1‐Luc group were measured every 2 days using digital calipers. Mice were euthanized when tumors reached 1000 mm^3^. In the CT26‐Luc re‐challenged mice, some mice that demonstrated clear signs of tumor growth were euthanized before reaching clinical endpoints, and their spleens were collected for ELISPOT analysis like before. In a separate study, the mice were provided CPMV adjuvant therapy and CT26‐Luc challenge like before. The survivors were then re‐challenged with 100000 CT26‐Luc s.c. The tumors were measured every 2 days until clinical endpoints.

### Adjuvant Therapy in Pre‐Metastatic Mice

To simulate the presence of metastatic lesions already present within the i.p. space, BALB/C mice were first injected with 50000 CT26‐Luc cells i.p. 3 days later, 200 µg of CPMV or PBS were injected i.p. followed by injection of 500000 CT26‐Luc cells after 7 days. The circumference and weight of the mice were measured every 2 days.

### Longitudinal Analysis of CPMV Prophylaxis i.p

The longitudinal ability of CPMV to protect against CT26‐Luc challenge was carried out by incrementally increasing the time until CT26‐Luc i.p. tumor challenge. Mice were injected with 200 µg of CPMV and then with 500000 CT26‐Luc cells 7, 14, 21, and 28 days following CPMV injection.

To analyze CPMV biodistribution and clearance from the i.p. space, the i.p. fluid as well as the spleen, liver, kidneys, and lungs were collected after 1 and 2 weeks. Total RNA was extracted and purified from the solid organs using RNAqueous Total RNA Isolation Kit (Invitrogen) according to manufacturer's instructions. RNA from i.p. fluid was extracted using QIAamp Viral RNA mini kit (Qiagen) according to manufacturer's instructions.

Five nanograms of total RNA extracted from organs and i.p. fluid was probed for CPMV RNA using TaqMan Fast Virus 1‐Step Master Mix (Applied Biosystems). Serial dilutions (100, 10, 1, 0.1, and 0.01 ng CPMV RNA) were used as templates to graph a standard curve. All experiments included a no template control (NTC). CPMV Primers were designed and purchased from Integrated DNA Technologies:

Probe 5′‐/56FAM/TCGGGTTGT/ZEN/TGTTTGATGTTGGCC/3IABkFQ/−3′

Primer 1 (RV) 5′‐CAT GGA GTC TTG AGA GCA GAT AG‐3′

Primer 2 (FW) 5′‐ACA GCT ACC ACC AAC ATT TCT‐3′

This experiment was performed using a CFX96 touch real‐time PCR detection system (BioRad).

### Administration Route Analysis and Mechanism of Action of CPMV Prophylaxis Against CT26‐Luc i.p

To determine whether CPMV had to be injected locally i.p. to protect against CT26‐Luc, the route of administration was altered prior to CT26‐Luc challenge. Two hundred micrograms of CPMV was injected i.p., intravenously (i.v.), or s.c. One week following CPMV administration, the mice were challenged with 500000 CT26‐Luc cells i.p., and the tumor growth and survival were monitored.

In a separate experiment, CPMV was injected i.p., i.v., and s.c. as above. One week following injection, the i.p. fluid was collected with i.p. gavage, and the collected cells were readied for flow cytometry analysis. The cells were stained with a 1:1000 dilution of a LIVE/DEAD Aqua (Thermo Scientific) solution and incubated at RT for 20 min followed by washing with PBS. The cells were then fixed for 20 min with 2% (v/v) PFA. Following washing, the F_c_ regions of the cells were blocked with an α‐CD16/32 blocking antibody (Biolegend) for 20 min at 4 °C. The cells were stained with the following antibodies (all from Biolegend) at a 1:200 dilution for 1 h at 4 °C: Pacific Blue – CD45; APC‐Cy7 – Ly6G; PE‐Cy7 – Ly6C; SuperBright 645 – CD11b; Brilliant Violet 605 – NKp46; PE – F4/80; PerCP‐Cy5.5 – CD86; APC – MHCII; SuperBright 780 – CD11c. Following washing, the cells were stored at 4 °C in FACS buffer (PBS + 0.5 m EDTA + FBS + 1 m HEPES) until use. Flow cytometry was carried out on a FACSCelesta, and data analysis was implemented using FlowJo.

The i.p. fluid was also analyzed via MSD using a customizable U‐PLEX analyzing the following cytokines/chemokines according to manufacturer's instructions: GM‐CSF, IFNγ, IL‐2, IL‐4, IL‐5, IL‐10, IL‐12p70, IL‐13, KC/GRO, eotaxin, IFN‐β, IL‐9, IL‐15, IP‐10, MCP‐1, MIP‐1α, MIP‐1β, TNF‐α, VEGF‐A, IFN‐α, IL‐12/IL‐23p40, IL‐17A, and RANTES. The plate was read using a MESO QuickPlex SQ 120 instrument and analyzed using MSD Workbench 4.0 software.

### Tandem Mass Tag (TMT)

TMT was carried out with the help of the Biomolecular and Proteomics Mass Spectrometry Facility at UC San Diego. The protein fractions following i.p. injection of CPMV were first digested through pelleting of the samples. The pellet was resuspended in 600 µl of 8 m urea made in 100 mm Tris pH 8.0 and by vortexing for 5–10 min. TCEP reducing agent was added to a final concentration of 10 mm. The samples were then frozen ON at −20 °C. The next day, the solution was thawed and vortexed for another 5 min until the solution became clear. Chloro‐acetamide solution was added to a final concentration of 40 mm and vortexed for 5 min. An equal volume of 50 mm Tris pH 8.0 was added to the sample to reduce the urea concentration to 4 m followed by the addition of Lys C at a 1:500 ratio of LysC to protein content. The solution was incubated at 37 °C in a rotating incubator for 4–6 h, and 50 mm Tris pH 8.0 was added to reduce the urea concentration to 2 m. Trypsin was added at a 1:50 ratio of trypsin to protein content and incubated ON. The next day, the solution was acidified using TFA to a final concentration of 0.5% (v/v) TFA and vortexed for 5 min. The sample was centrifuged at 15700 x g for 5 min to obtain aqueous and organic phases. The lower aqueous phase was collected and desalted using 100 mg C18‐StageTips as described by the manufacturer's protocol. The peptide concentration of the sample was measured using BCA after resuspension in iTRAQ dissolution buffer.

Four tags of Thermo Scientific TMTpro 16plex (Catalog number: A52045) was used for the labeling of the samples. The labeling protocol was followed according to the manufacturer's instructions. The CPMV i.p. sample was tagged with TMT16‐133N, and the PBS i.p. sample was tagged with TMT16‐134N. Pierce High pH Reversed‐Phase Peptide Fractionation Kit (Catalog number: 84 868) was used for the high pH fractionation according to the manufacturer's instructions with the exception that 12 fractions were generated.

Each fraction was analyzed by ultra‐high‐pressure liquid chromatography (UPLC) coupled with tandem mass spectroscopy (LC‐MS/MS) using nano‐spray ionization. The nano‐spray ionization experiments were performed using an Orbitrap fusion Lumos hybrid mass spectrometer (Thermo Fisher) interfaced with nano‐scale reversed‐phase UPLC (Thermo Dionex UltiMate™ 3000 RSLC nano System) using a 25 cm, 75 µm ID glass capillary packed with 1.7 µm C18 (130) BEH^TM^ beads (Waters Corporation). Peptides were eluted from the C18 column into the mass spectrometer using a linear gradient (5–80%) of ACN at a flow rate of 375 µl min^−1^ for 120 min. The buffers used to create the ACN gradient were: Buffer A (98% H_2_O, 2% ACN, 0.1% formic acid) and Buffer B (100% ACN, 0.1% formic acid). Mass spectrometer parameters are as follows: an MS1 survey scan using the orbitrap detector (mass range (m/z): 400–1500 using quadrupole isolation, 60 000 resolution setting, spray voltage of 2200 V, ion transfer tube temperature of 275 °C, AGC target of 400 000, and maximum injection time of 50 ms) was followed by data dependent scans at which the top speed for the most intense ions with charge states set to only include +2‐5 ions, 5 s exclusion time, and while selecting ions with minimal intensities of 50 000 at in which the collision event was carried out in the high energy collision cell (HCD Collision Energy of 38%). The first quadrupole isolation window was set at 0.7 (m/z). The fragment masses were analyzed in the Orbi‐trap mass analyzer with mass resolution setting of 15 000 and the following parameters: ion trap scan rate of turbo, first mass m/z of 100, AGC Target 20 000 and maximum injection time of 22 ms). Protein identification and quantification was carried out using Peaks Studio 8.5 (Bioinformatics Solutions Inc.).

### ID8‐Defb29/Vegf‐A‐Luc Prophylaxis with CPMV

To demonstrate the broad applicability of CPMV prophylaxis in multiple tumor types, an ID8‐*Defb29/Vegf‐A‐*Luc ovarian cancer murine model was also tested. 6‐ to 8‐week‐old female C57BL/6J mice were purchased and injected with 200 µg of CPMV i.p. followed by injection of 2 × 10^6^ ID8‐*Defb29/Vegf‐A*‐Luc i.p. Mice were euthanized when the body weight reached >75% of the original body weight or the body weight totaled over 30 g. Mice were also euthanized when the abdominal circumference reached >60% of the original circumference or reached a circumference of 9 cm. The circumference and body weight were used as indicators of tumor growth following i.p. tumor dissemination and ascites development. At day 67, the spleens of the surviving mice were collected and analyzed by ELISPOT. The splenocytes were stimulated with either media only, 500000 B16F10 cells, 500000 ID8‐*Defb29/Vegf‐A*‐Luc cells, or PMA/iono.

### CPMV Prophylaxis Against B16F10 Melanoma and 4T1‐Luc TNBC Metastasis to the Lungs

The ability of CPMV to protect against metastasis to the lungs was also investigated in a B16F10 murine i.v. model. Female 6–8 week old C57BL/6J mice were immunized with 200 µg of CPMV s.c., i.v., and i.p followed by injection of 100000 B16F10 cells i.v. After 3 weeks, the lungs were harvested from the mice and the tumor nodules were manually counted. In a comparative study, CPMV was investigated head‐to‐head against other potential adjuvants and immunomodulatory small molecules. Mice were injected i.p. or i.v. with either 200 µg of CPMV or CCMV, or 5 µg of diABZI or imiquimod. 100000 B16F10 cells were injected i.v., and the lungs were harvested and tumor nodules were manually counted after 2 weeks. The immunogenicity of the CPMV and other reagents was also compared using the RAW264.7‐Luc reporter line as indicated above.

For the 4T1‐Luc prophylaxis studies, 6–8 week old female BALB/C mice were purchased. They were injected i.p. with 200 µg of CPMV followed by the injection of 100000 4T1‐Luc cells i.v. The growth of the 4T1‐Luc cells was monitored using an IVIS (Xenogen) every 2–4 days. To measure luminescence, 200 µL of a 15 mg mL^−1^ solution of D‐luciferin (Gold Biotechnologies) was injected i.p, and the luminescence was measured using ROI measurements on the Living Image 3.0 software. The weight of the mice and luminescence within the lungs were measured every 2 days. Three weeks following tumor inoculation, the lungs were collected and stored in Bouin's solution (Sigma Aldrich) ON followed by storage in 70% (v/v) EtOH. The tumor nodules were then manually counted.

### Mechanism of Action of CPMV Protection Against Lung Metastatic Tumor Nodules

The sera of C57BL/6J mice was isolated from peripheral blood and collected for MSD analysis like above. The sera was also collected for flow cytometry analysis. The blood was incubated with 10 mL of 1x RBC lysis buffer for 5 min at RT followed by the deactivation of the lysis buffer with 20 mL of PBS. Cells were subjected to the same protocol as with the i.p. fluid flow cytometry analysis except that instead of a Brilliant Violet 605 NKp46 antibody, a Brilliant Violet 605 NK1.1 antibody was used. The flow experiments were also repeated in the spleens and lungs.

### Surgical Removal of 4T1‐Luc Followed by CPMV Adjuvant Therapy

To ascertain that CPMV was effective in a more clinically‐relevant model, 100000 4T1‐Luc TNBC cells were injected s.c. in the left flank of female BALB/C mice and surgically removed after two weeks. The mice were given anesthetic lidocaine (Vet One) according to the manufacturer's instructions. The following day, the mice were injected i.v. with 200 µg of CPMV or 200 µL of PBS. The metastasis and recurrence of the 4T1‐Luc cells were monitored using an IVIS. The mice weight was also measured, and mice were sacrificed when their weight decreased more than 15% from their heaviest recorded weight. Weight loss was used as an indicator of lowered quality of life in the mice due to tumor burden with 15% being the clinical endpoint according to IACUC regulations.

### Statistical Analysis

All statistical analyses were carried out using GraphPad Prism. When comparing between multiple groups such as when comparing tumor nodule counts, one‐way ANOVA was utilized. When only two groups were present, a Student's T‐test was employed. A comparative analysis with time utilized a two‐way ANOVA, and survival curves were analyzed using a log‐rank (Mantel Cox) test. All graphs were showing the mean ± SD, and the number of samples included in each statistical analysis was written out within the figure captions. A p‐value of < 0.05 was deemed significant.

## Conflict of Interest

Dr. Steinmetz is a co‐founder of, has equity in, and has a financial interest in Mosaic ImmunoEnginering Inc. Dr. Steinmetz is a co‐founder of, and serves as manager of Pokometz Scientific LLC, under which she is a paid consultant to Mosaic ImmunoEngineering Inc., Flagship Labs 95 Inc., and Arana Biosciences Inc. The other authors declare no potential COI.

## Supporting information

Supporting Information

## Data Availability

The data that support the findings of this study are available from the corresponding author upon reasonable request.
