# Supplementary material for: Systemic Administration of Cowpea Mosaic Virus Demonstrates Broad Protection Against Metastatic Cancers
Source: Adv Sci (Weinh). 2024 Mar 2;11(18):2308237. doi: 10.1002/advs.202308237 (PMC11095214; doi:10.1002/advs.202308237)
Supplement: Supplementary file 1 — Supporting Information [file ADVS-11-2308237-s001.pdf]

ADVANCED SCIENCE
Open Access

Supporting Information

for *Adv. Sci.*, DOI 10.1002/advs.202308237

Systemic Administration of Cowpea Mosaic Virus Demonstrates Broad Protection Against Metastatic Cancers

*Young Hun Chung, Zhongchao Zhao, Eunkyeong Jung, Anthony O. Omole, Hanyang Wang, Lucas Sutorus and Nicole F. Steinmetz**

Supplementary Materials for

Systemic Administration of Cowpea Mosaic Virus Demonstrates Broad Protection Against Metastatic Cancers

Young Hun Chung[1,2], Zhongchao Zhao[2,3,4], Eunkyeong Jung[3], Anthony O. Omole[3], Hanyang Wang[5], Lucas Sutorus[3], Nicole F. Steinmetz[1,2,3,4,6,7,8*]

Corresponding author: nsteinmetz@ucsd.edu

[1]Department of Bioengineering, University of California, San Diego, La Jolla, CA, USA.
[2]Moores Cancer Center, University of California, San Diego, La Jolla, CA, USA.
[3]Department of NanoEngineering, University of California, San Diego, La Jolla, CA, USA.
[4]Center for Nano-Immuno Engineering, University of California, San Diego, La Jolla, CA, USA.
[5]Department of Biology, University of California, San Diego, La Jolla, CA, USA.
[6]Department of Radiology, University of California, San Diego, La Jolla, CA, USA.
[7]Institute for Materials Discovery and Design, University of California, San Diego, La Jolla, CA, USA.
[8]Center for Engineering in Cancer, University of California, San Diego, La Jolla, CA, USA.

The PDF file includes:

Figs. S1 to S14

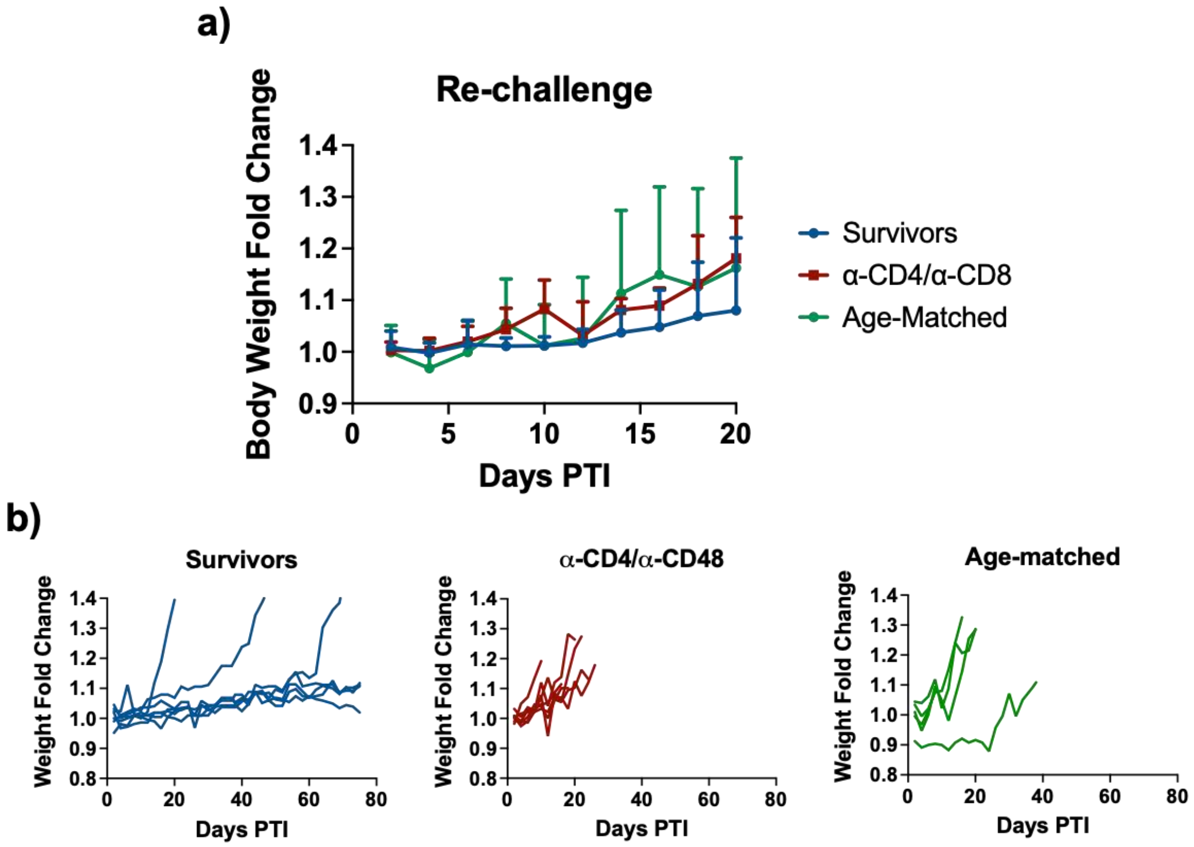

Fig. S1. Weight of mice following re-challenge. a) Fold change in weight of mice compared to their weights at the time of re-challenge. The weight of the mice from the re-challenge experiment in Figure 2e-g were measured every two days. b) The individual weight fold changes from Fig. S1a. The data in a) is the average weight fold change, and the error bars represent the standard deviation (n = 5-7).

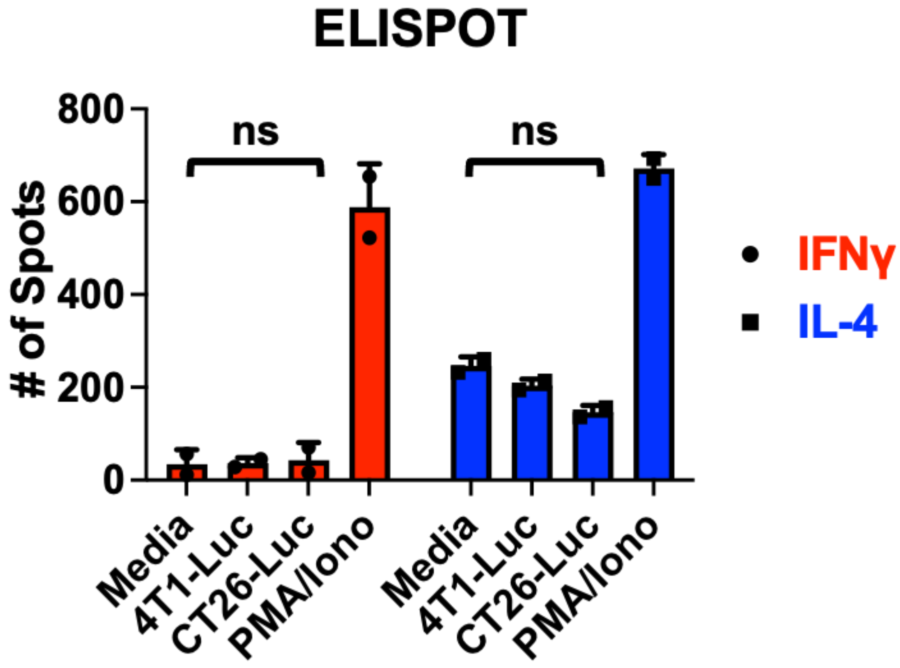

Fig. S2. ELISPOT on surviving mice that did not survive re-challenge. Two mice that were re-challenged with CT26-Luc, but showed clear signs of tumor growth, were sacrificed before clinical endpoints and their splenocytes were used for ELISPOT analysis. While splenocytes from mice that survived re-challenge (Fig. 2h, i) demonstrated potent IFNγ production against CT26-Luc cells, these mice did not demonstrate significant IFNγ production indicating loss of IFNγ-producing splenocytes and thereby the importance of the memory T cell response in the clearance of re-challenged i.p.-disseminated tumors. The data is showing the average number of spots, and the error bars are showing the standard deviation (n = 2). The data was analyzed using Student's T-test. ns = not significant.

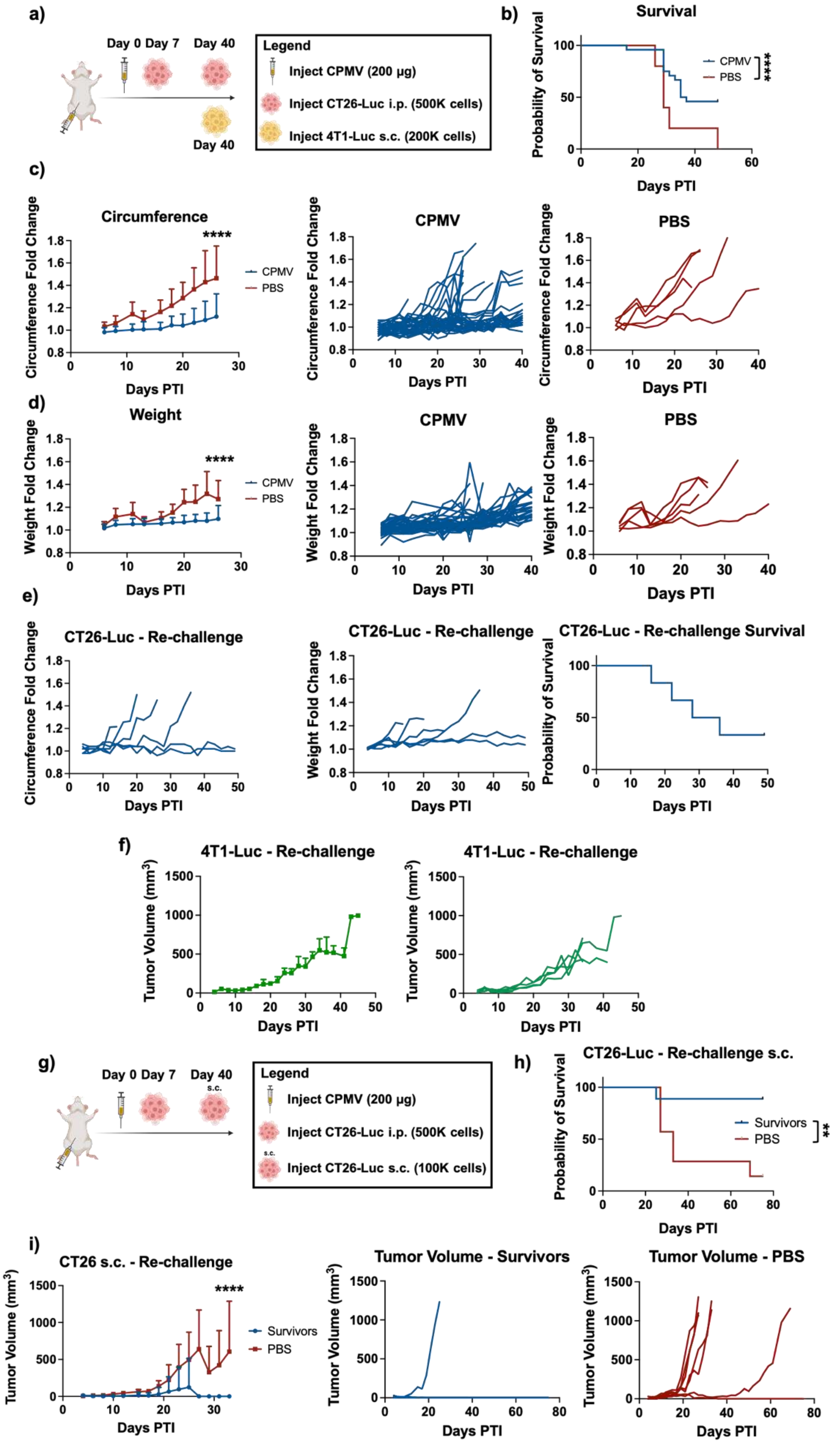
a)
Day 0 Day 7 Day 40
Day 40
Legend
Inject CPMV (200 µg)
Inject CT26-Luc i.p. (500K cells)
Inject 4T1-Luc s.c. (200K cells)
b)
Survival
Probability of Survival
CPMV
PBS
****
Days PTI
c)
Circumference
Circumference Fold Change
****
CPMV
PBS
Days PTI
CPMV
PBS
d)
Weight
Weight Fold Change
****
e)
CT26-Luc - Re-challenge
CT26-Luc - Re-challenge Survival
f)
4T1-Luc - Re-challenge
Tumor Volume (mm³)
g)
Day 0 Day 7 Day 40
s.c.
Legend
Inject CPMV (200 µg)
Inject CT26-Luc i.p. (500K cells)
Inject CT26-Luc s.c. (100K cells)
h)
CT26-Luc - Re-challenge s.c.
Survivors
PBS
**
i)
CT26 s.c. - Re-challenge
Survivors
PBS
Tumor Volume - Survivors
Tumor Volume - PBS

Fig. S3. Formation of immune memory is tumor specific, but tissue agnostic. a) Injection schedule of CT26-Luc and 4T1-Luc re-challenge. BALB/C mice were injected with CPMV (n = 40) prophylactically followed by CT26-Luc injection i.p. Survivors were re-challenged with CT26-Luc i.p. or 4T1-Luc s.c. Survival curve b), circumference fold change c), and weight fold change d) following initial challenge after CPMV prophylaxis. The results closely mirror the data in Fig. 2b-d. e) Circumference fold change (left), weight fold change (middle), and survival of mice following re-challenge with CT26-Luc i.p. (n = 6). The results closely mirror the data from Fig. 2e-g. f) Tumor volume curve (left) and individual tumor growth curves (right) of mice (n = 5) following 4T1-Luc s.c. re-challenge. There were no long-term survivors, and all tumors grew unabated indicating that immune memory is tumor specific. Survival of the mice can be found on Fig. 2j. g) Injection schedule of CT26-Luc i.p. followed by CT26-Luc re-challenge s.c. to demonstrate that immune memory is tissue agonistic. h) Survival and i) tumor growth curves following CT26-Luc s.c. re-challenge. The initial circumference c) and weight fold changes d) as well as the tumor volume curves i) were analyzed with two-way ANOVA. The data is showing the average circumference and weight fold changes, and the tumor volume, and the error bars represent the standard deviation. The survival curves b) and h) were analyzed with a Log-rank (Mantel-Cox) test. ** = $p < 0.01$, **** = $p < 0.0001$. The schematics were created on Biorender.com.

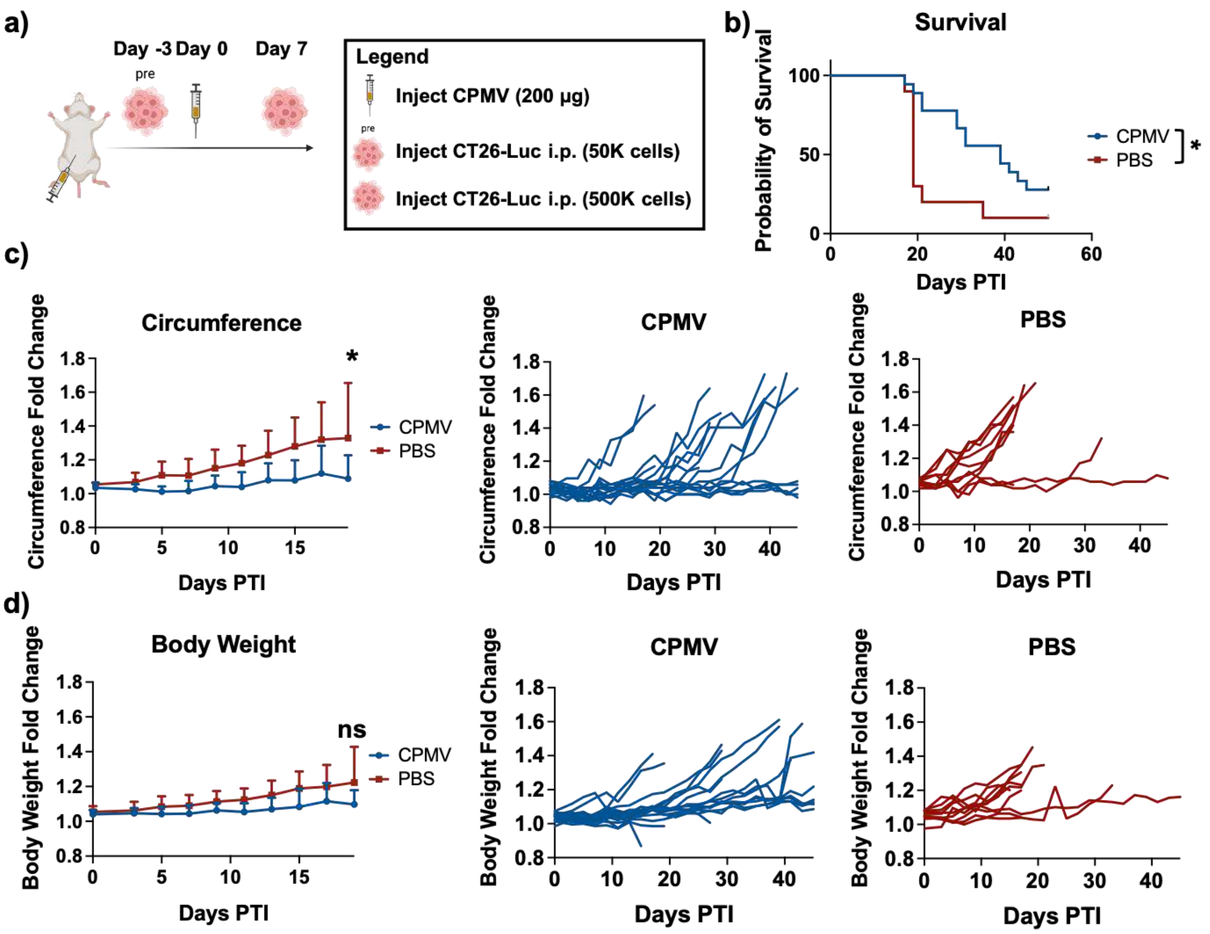

Fig. S4. CPMV adjuvant therapy in pre-metastatic mice. a) Injection schedule. Female BALB/C mice are injected with 50,000 CT26-Luc cells i.p. prior to CPMV adjuvant treatment (n = 18) followed by injection of 500,000 CT26-Luc cells i.p. to stimulate patients with pre-disseminated disease. b) Survival curve of mice following CPMV adjuvant treatment. c) Circumference fold change of all mice (left), individual curves in the CPMV group (middle), and individual curves in the PBS group (right). d) Weight fold changes of all mice (left), individual curves in the CPMV group (middle), and individual curves in the PBS group (right). The circumference and body weight graphs were analyzed using two-way ANOVA, and the survival curve was analyzed using a log-rank (Mantel-Cox) test. The data represents the average values, and the error bars represent the standard deviation. * = $p < 0.05$, ns = not significant.

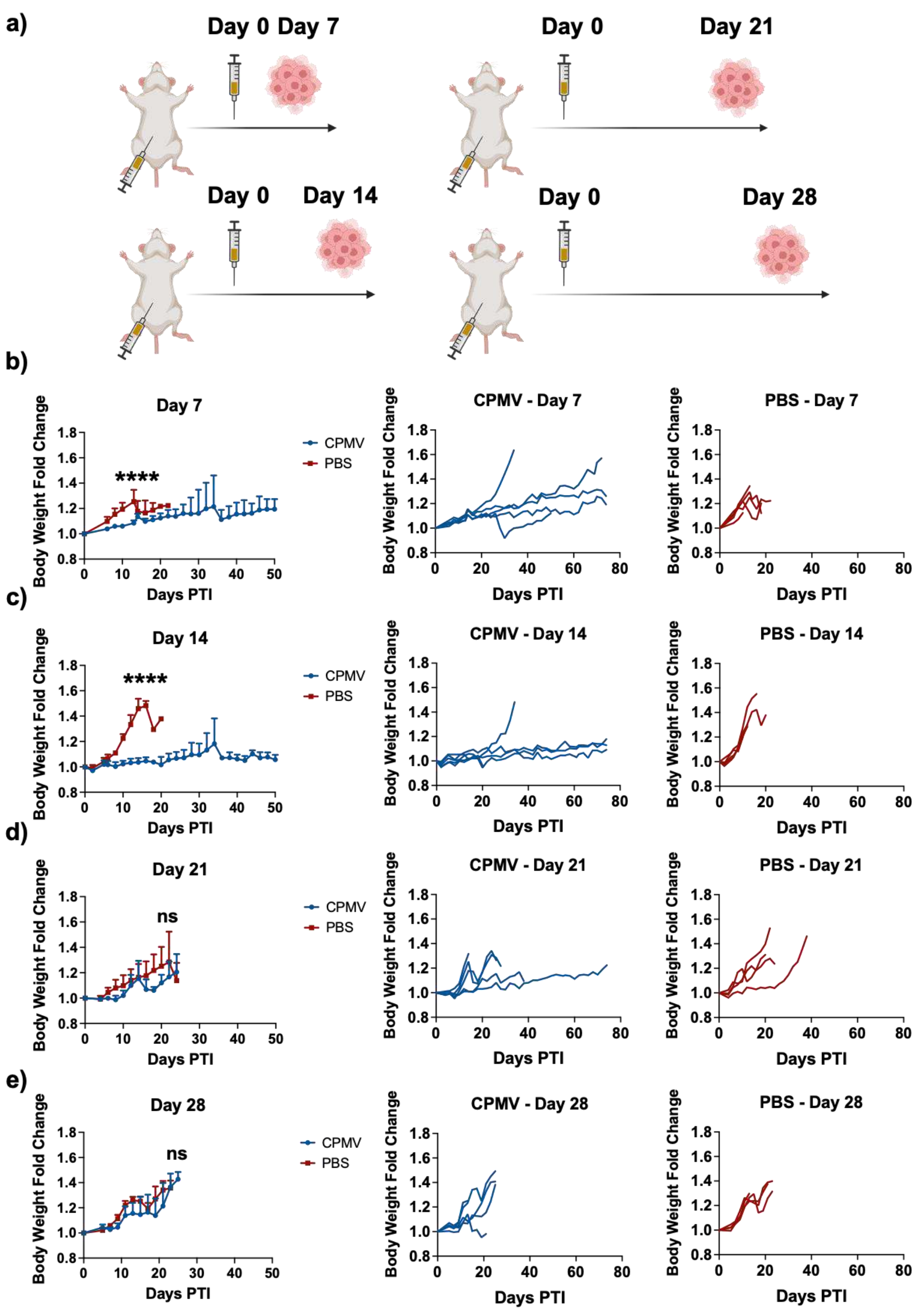

Fig. S5. Longitudinal analysis of body weight measurements in mice challenged with CT26-Luc after CPMV injection. a) Injection schedule. BALB/C mice (n = 5) were injected initially with CPMV followed by injection with 500,000 CT26-Luc cells i.p. after 7, 14, 21, and 28 days. b-e) Body weight fold change after injection of CPMV following CT26-Luc for b) 7 days, c) 14 days, d) 21 days, and e) 28 days. The body weight statistics were analyzed using two-way ANOVA. The curves represent the average body weigh fold change, and the error bars represent the standard deviation. **** = $p < 0.0001$, ns = not significant. The schematic in a) was created on Biorender.com.

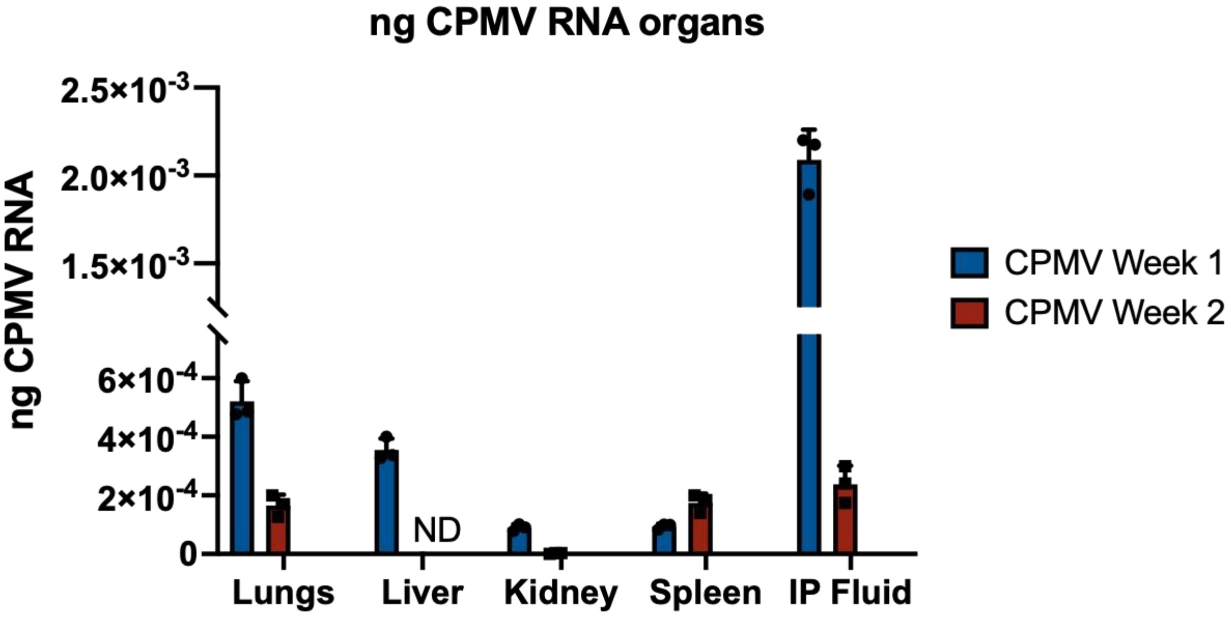

Fig. S6. Biodistribution and clearance of CPMV. CPMV biodistribution and clearance 1 and 2 weeks after i.p. injection were measured using qRT-PCR. CPMV biodistribution was investigated within the lungs, liver, kidney, spleen, and i.p. fluid, and normalized to mice injected with PBS (data not shown). CPMV is stable for extended periods of time within the i.p. space and was detectable up to 2 weeks after i.p. injection further demonstrating the long-lasting efficacy of CPMV after only a single injection. The data is showing the average ng of CPMV RNA, and the error bars represent the standard deviation (n = 3), ND = not detectable.

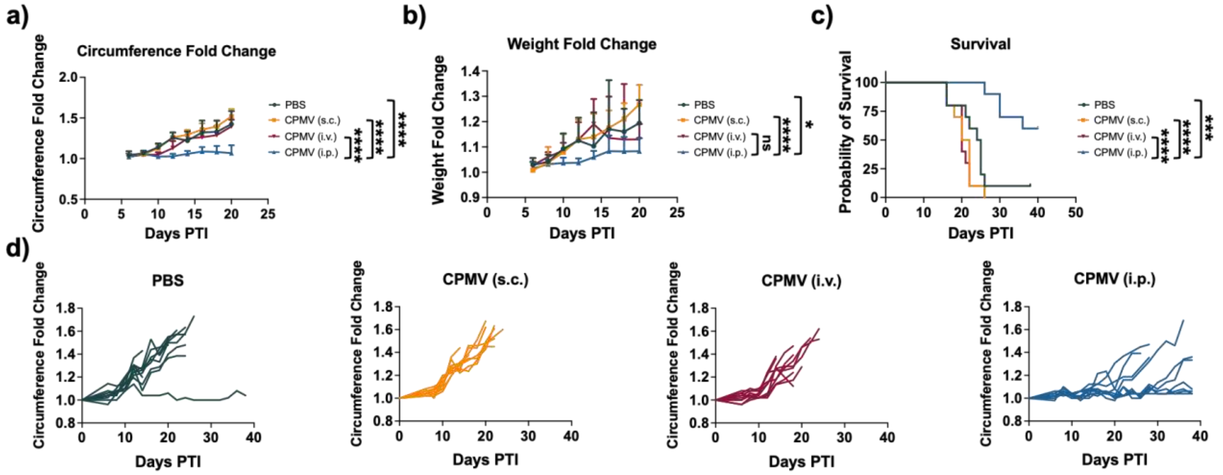

Fig. S7. Route of administration of CPMV in CT26-Luc i.p. protection. a) Circumference fold change in BALB/C mice injected with PBS, CPMV s.c., CPMV i.v., and CPMV i.p. (n = 10). b) Weight fold change. c) Survival of mice. d) Individual circumference growth curves in mice injected with PBS (left), CPMV s.c. (left, middle), CPMV i.v. (right, middle), and CPMV i.p. (right). Only CPMV injected i.p. showed significant benefit in reducing tumor growth and extending survival. The circumference and weight fold change graphs were analyzed using two-way ANOVA. The values represent the average circumference and weight fold changes, and the error bars represent the standard deviation The survival curve was analyzed using a log-rank (Mantel-Cox) test. * = $p < 0.05$, *** = $p < 0.001$, **** = $p < 0.0001$, ns = not significant.

a)

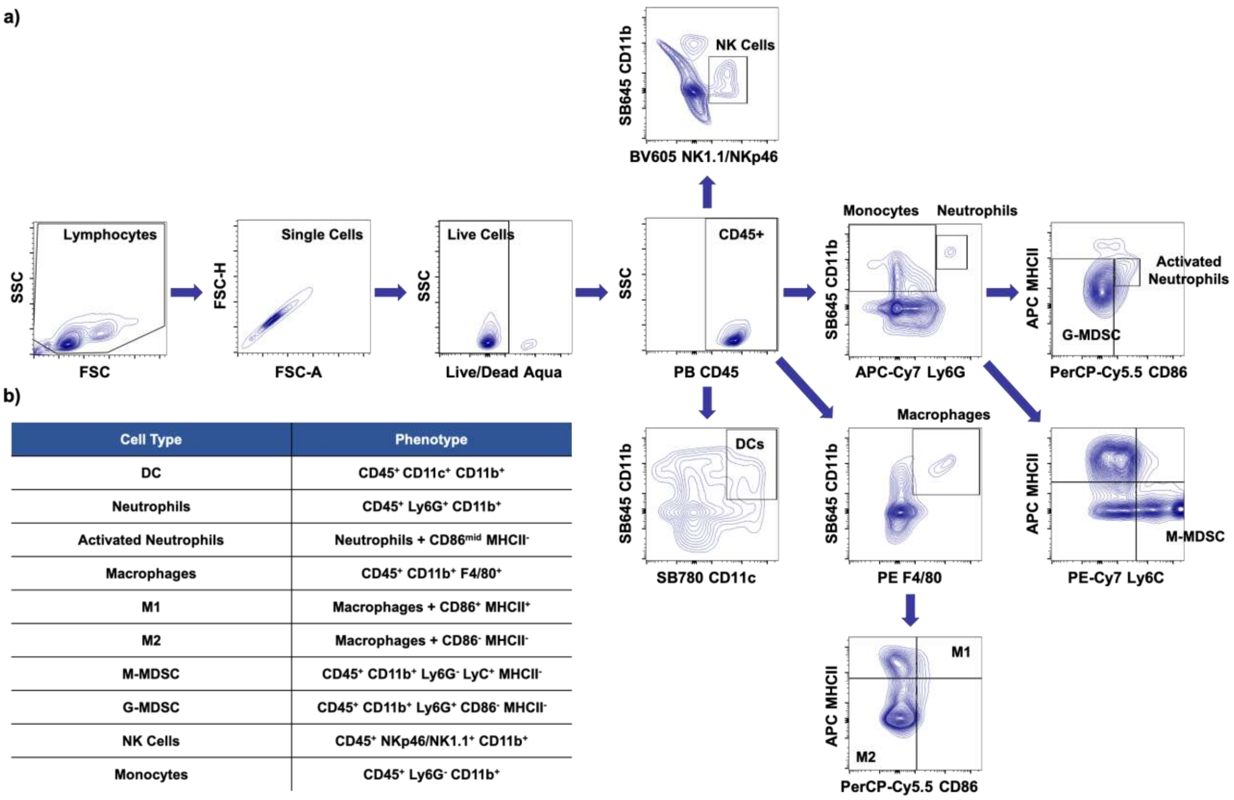

b)

Cell Type	Phenotype
DC	$CD45^+$ $CD11c^+$ $CD11b^+$
Neutrophils	$CD45^+$ $Ly6G^+$ $CD11b^+$
Activated Neutrophils	Neutrophils + $CD86^{mid}$ $MHCII^-$
Macrophages	$CD45^+$ $CD11b^+$ $F4/80^+$
M1	Macrophages + $CD86^+$ $MHCII^+$
M2	Macrophages + $CD86^-$ $MHCII^-$
M-MDSC	$CD45^+$ $CD11b^+$ $Ly6G^-$ LyC^+ $MHCII^-$
G-MDSC	$CD45^+$ $CD11b^+$ $Ly6G^+$ $CD86^-$ $MHCII^-$
NK Cells	$CD45^+$ $NKp46/NK1.1^+$ $CD11b^+$
Monocytes	$CD45^+$ $Ly6G^-$ $CD11b^+$

Fig. S8. Flow cytometry gating strategy. a) Flow cytometry gating. All the flow cytometry was accomplished using a FACSCelesta, and analyzed using FlowJo. For NK cells, either NKp46 or NK1.1 was used as the phenotypic marker depending on the mouse strain of BALB/C or C57BL/6J, respectively. SSC = side scatter, FSC = forward scatter, FSC-H = forward scatter height, FSC-A = forward scatter area, CD = cluster of differentiation, PB = Pacific Blue, BV = Brilliant Violet, SB = SuperBright, NK = Natural Killer, APC = allophycocyanin, Cy7 = Cyanine7, MHC = Major Histocompatibility Complex, Cy5.5 = Cyanine 5.5, G-MDSC = granulocytic myeloid derived suppressor cell, M-MDSC = monocytic myeloid derived suppressor cell, PE = phycoerythrin, DC = dendritic cell. b) Table showcasing the phenotype used to analyze each cell.

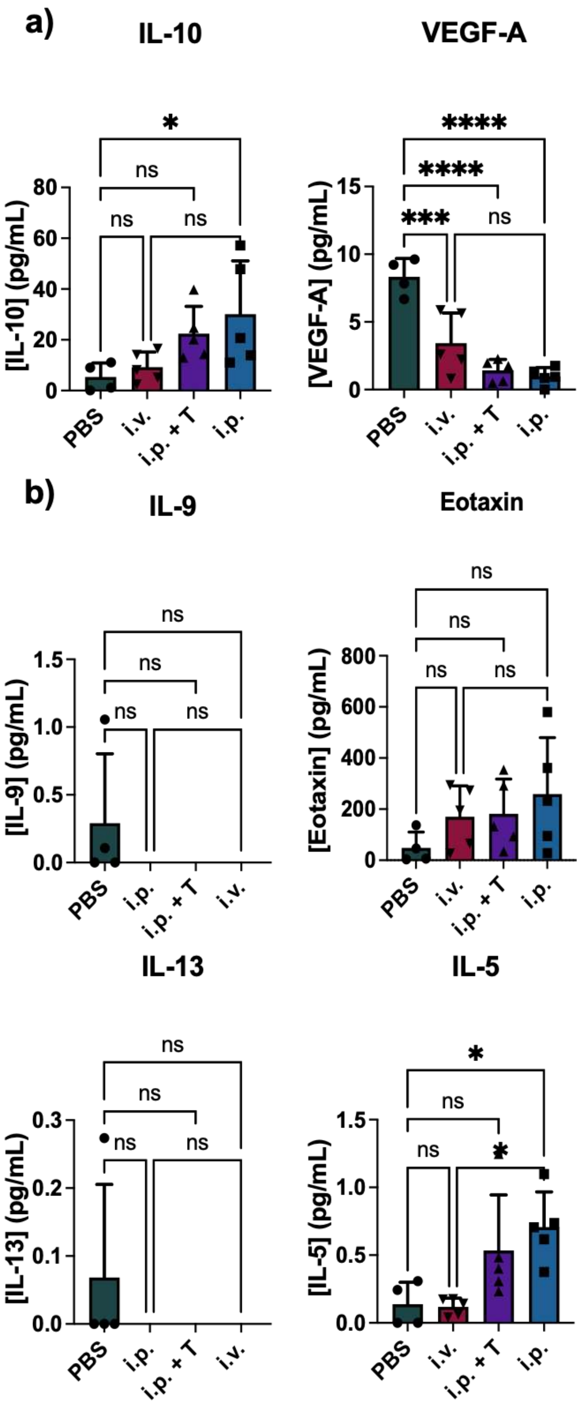

Fig. S9. MSD analysis of other cytokines not included in Fig. 4. a) MSD analysis of immunosuppressive cytokines (n = 4-5). b) MSD analysis of cytokines with both immunostimulatory and immunosuppressive functions or allergic reactions (n = 4-5). The groups were compared with one-way ANOVA. The data represents the average values, and the error bars represent the standard deviation. * = p < 0.05, *** = p < 0.001, **** = p < 0.0001, ns = not significant.

a)

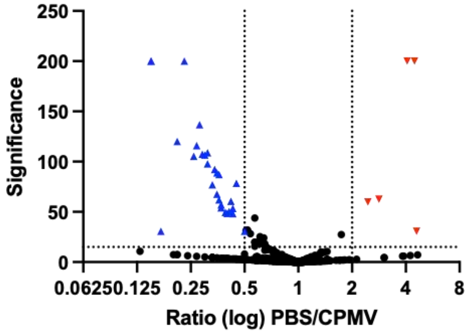

b)

Protein	Intensity	Significance
T-box transcription factor TBX22	2.3611E+06	10.97
Trinucleotide repeat-containing gene 18 protein	4.0382E+05	10.90
Immunoglobulin superfamily number 10	1.157E+06	10.93

c)

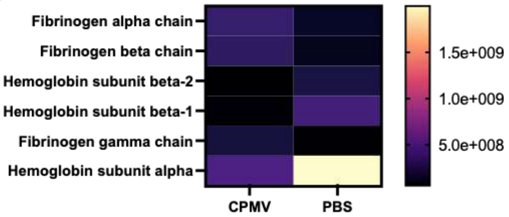

d)

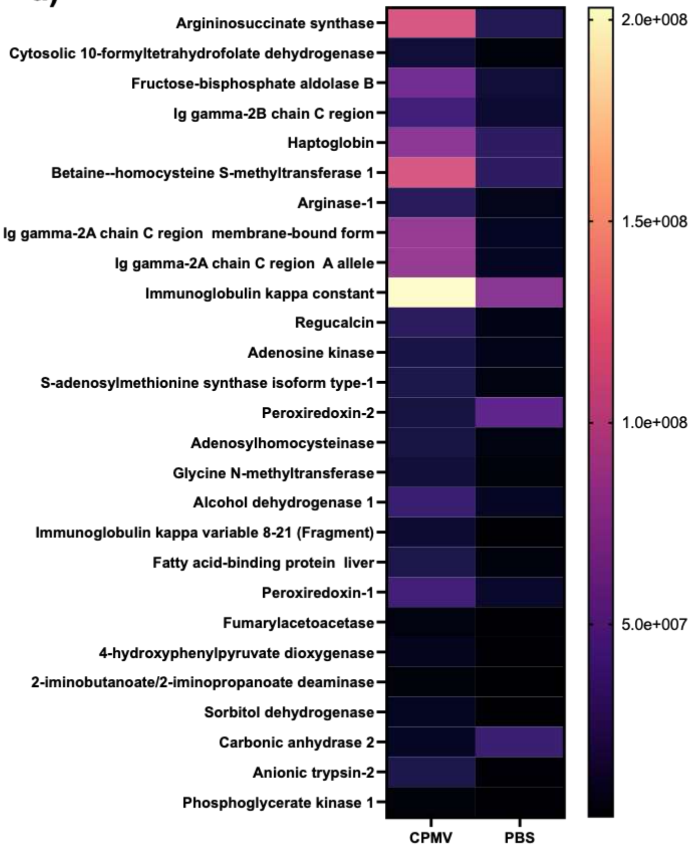

Fig. S10. Tandem mass tag (TMT) analysis of i.p. fluid following CPMV injection. a) Volcano plot of the identified proteins within the i.p. fluid comparing injection of PBS *vs.* CPMV one week after injection. Proteins were considered significant above a significance value of 15 and a ratio < 0.5 or > 2. A ratio < 0.5 and significance > 15 indicated that the proteins were upregulated following CPMV injection while a ratio > 2 indicates downregulation. b) Proteins that were found within the PBS samples, but not the CPMV samples are shown. These samples could not be graphed in a) due to the inability to calculate a ratio. c) Heat map of the intensities from fibrinogen and hemoglobin-related proteins. The intensities were much greater than that of the other proteins and so were plotted on a separate heat map. d) Heat map of the intensities of all the other significant proteins found within the i.p. fluid.

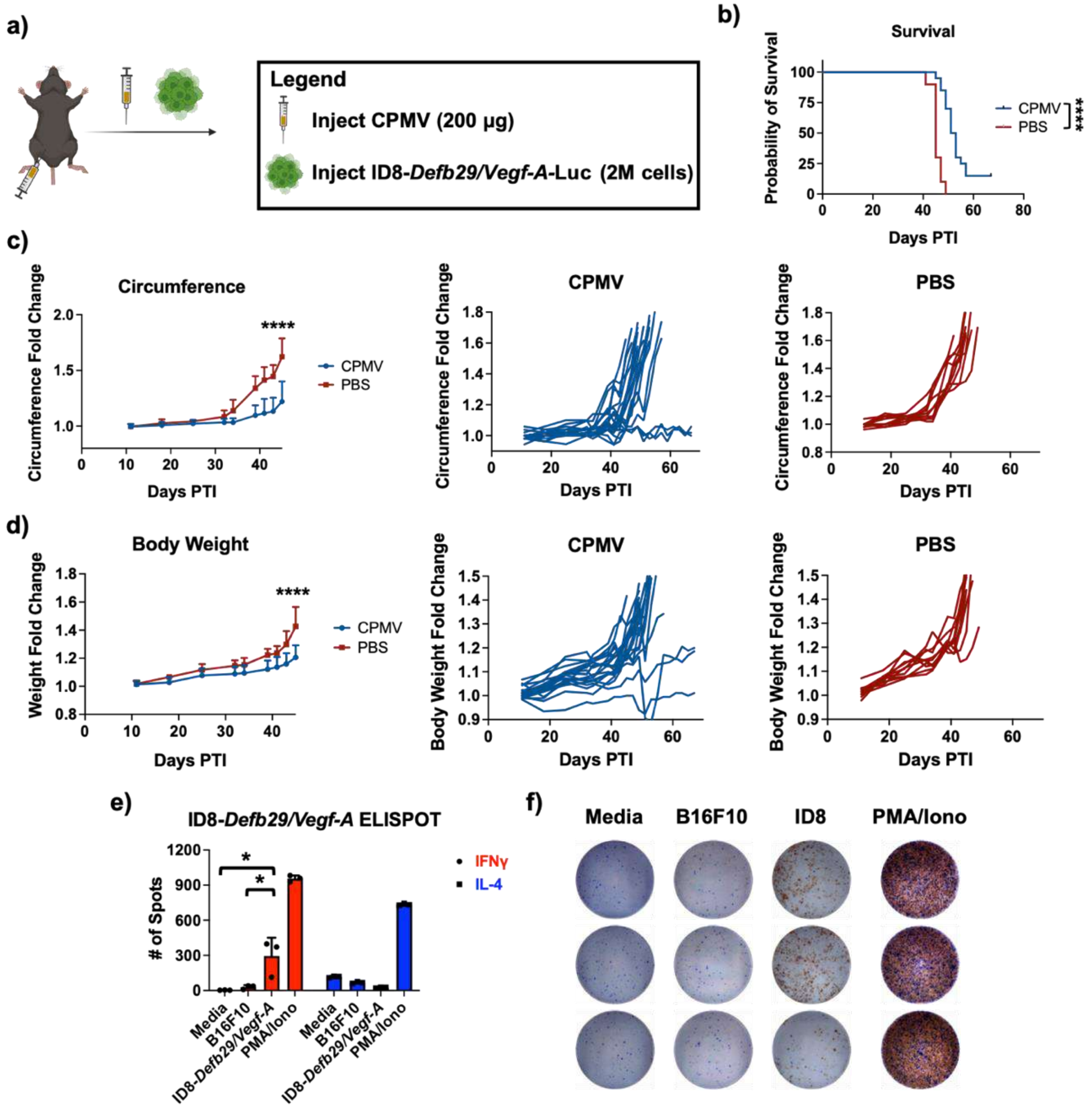

Fig. S11. CPMV adjuvant therapy against an ID8-*Defb29/Vegf-A*-Luc ovarian cancer model. a) Injection schedule. C57BL/6J mice (n = 20) were injected i.p. with 200 μg of CPMV followed by injection of 2 x 10^6 ID8-*Defb29/Vegf-A*-Luc cells i.p. after one week. b) Survival curve of mice. c) Circumference fold change of CPMV and PBS mice (left). Individual curves for CPMV (middle) and PBS (right) are also shown. d) Body weight fold change of CPMV and PBS mice (left). Individual curves for CPMV (middle) and PBS (right) are also shown. e) ELISPOT of the survivors in the CPMV group (n = 3). Splenocytes were harvested and stimulated with media only, B16F10 (negative control), ID8-*Defb29/Vegf-A*-Luc, and PMA/iono (positive control). f) Qualitative images of the ELISPOT data in e). The red spots are indicative of IFNγ production while the blue spots indicate IL-4 production corresponding to a Th1 and Th2 response, respectively. The survival curve was analyzed using a log-rank (Mantel-Cox) test, the circumference and body weight were analyzed using two-way ANOVA, and the ELISPOT data was analyzed using a Student's T-test. The data in c), d), and e) represent the average values, and the error bars represent the standard deviation. * = $p < 0.05$, **** = $p < 0.0001$. The schematic in a) was created on Biorender.com.

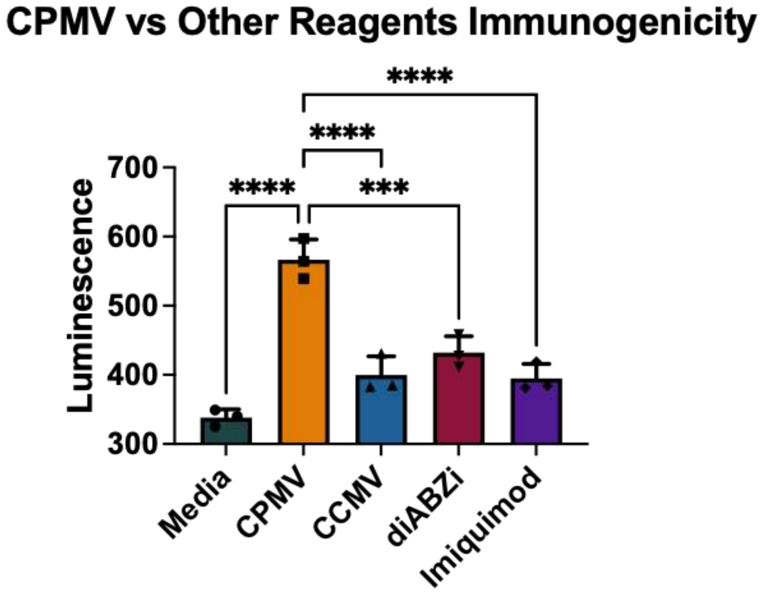

Fig. S12. RAW264.7-Luc NFκB signaling of CPMV and other adjuvants. 100,000 RAW264.7-Luc cells were incubated with 10 x 10^6 molecules of each reagent per cell and incubated for 24 h at 37°C. The luminescent output was measured using SteadyGlo (Promega) and read on a plate reader. The luminescence was analyzed using one-way ANOVA. The data represents the average luminescence, and the error bars represent the standard deviation. The readings were read in triplicate. *** = $p < 0.001$, **** = $p < 0.0001$. CCMV = cowpea chlorotic mottle virus.

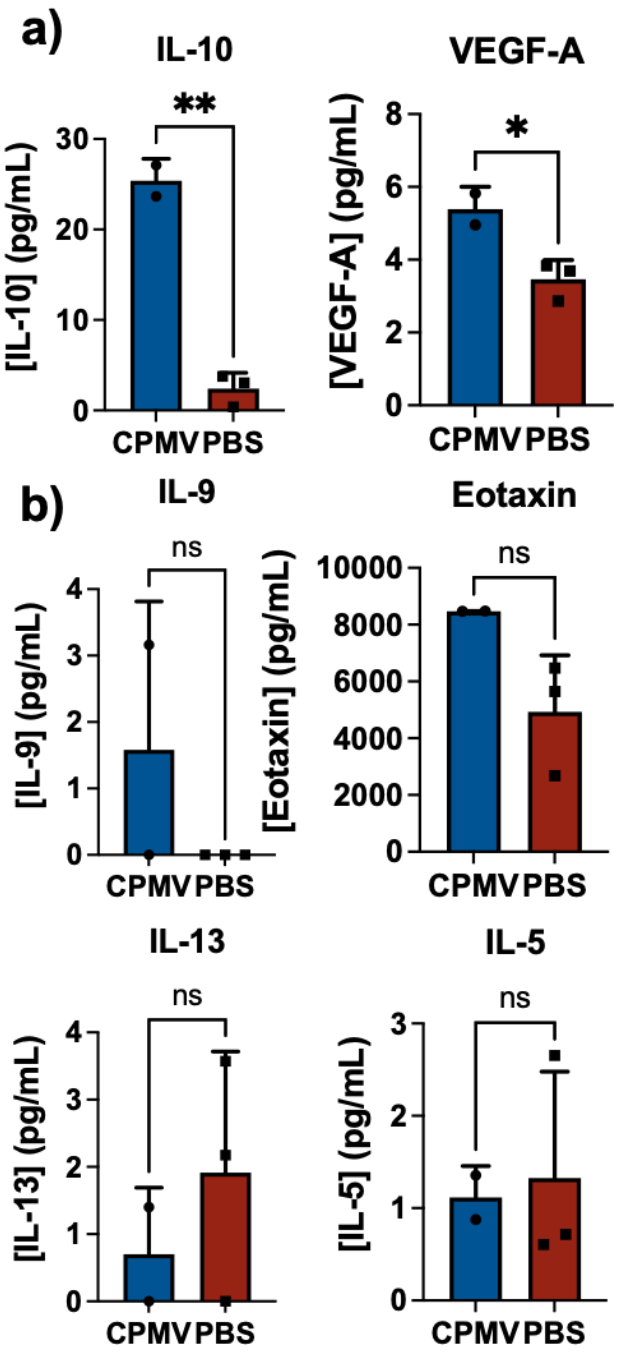

Fig. S13. MSD analysis of cytokines not shown in Fig. 6. a) MSD analysis of immunosuppressive cytokines (n = 2-3). b) MSD analysis of cytokines with both immunostimulatory and immunosuppressive functions or allergic reactions (n = 2-3). The groups were compared with Student's T-test. The values represent the average values, and the error bars represent the standard deviation. * = $p < 0.05$, ** = $p < 0.01$, ns = not significant.

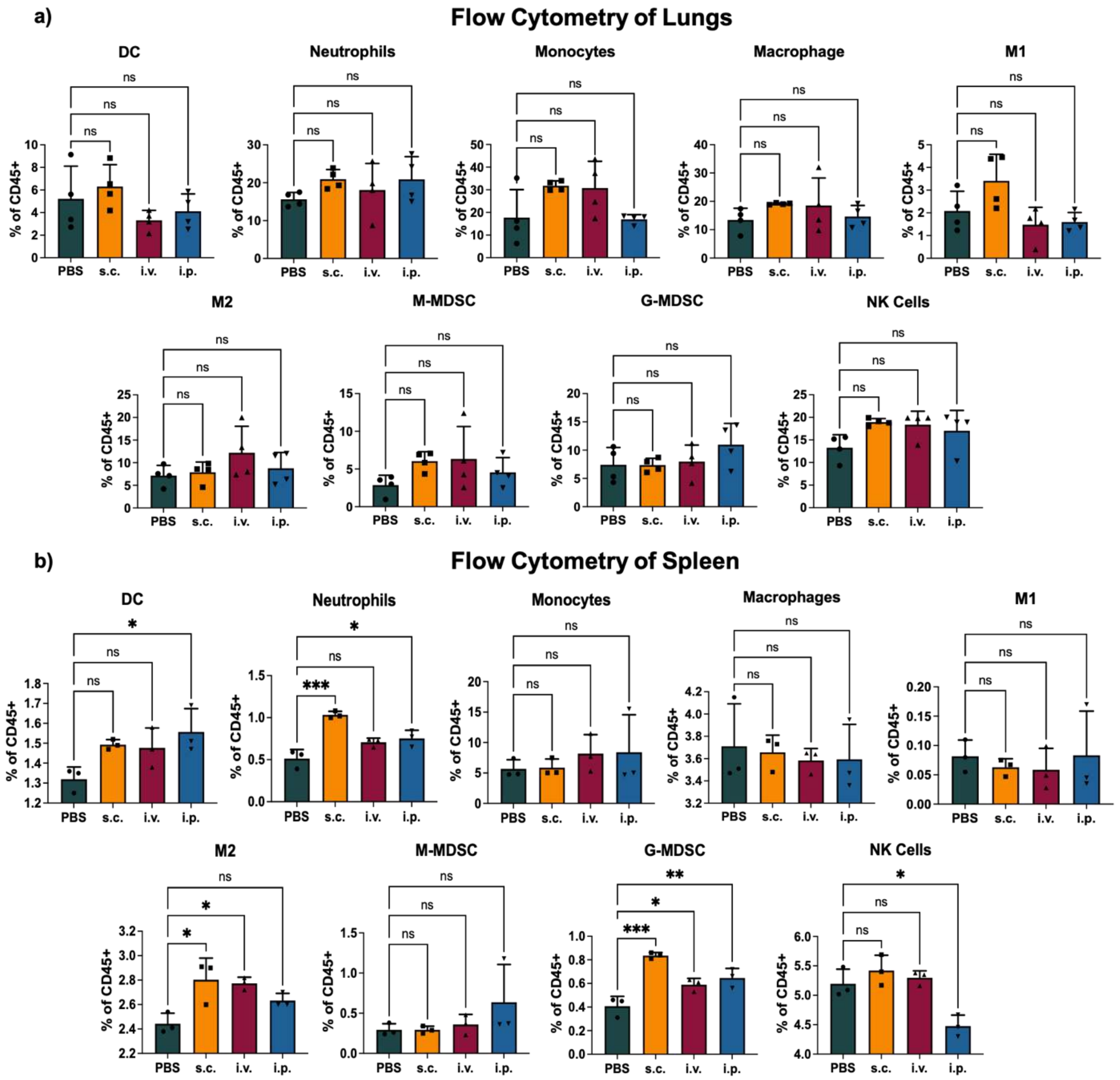

Fig. S14. Flow cytometry analysis of lungs and spleens following CPMV injection. a) Flow cytometry of the lungs. b) Flow cytometry of the spleen. Analyses in both the lungs and the spleen of the mice indicate minimal differences in the immune cell populations thereby demonstrating that clearance of the CTCs is most likely mediated by increased immune cell populations found within the blood of the mice as found in Fig. 6d. All samples in a) and b) were run in triplicate and were compared using one-way ANOVA. The values represent the average values, and the error bars represent the standard deviation. * = $p < 0.05$, ** = $p < 0.01$, *** = $p < 0.001$, ns = not significant.
